# The Coxsackievirus and Adenovirus Receptor (CAR) Undergoes Ectodomain Shedding and Regulated Intramembrane Proteolysis (RIP)

**DOI:** 10.1371/journal.pone.0073296

**Published:** 2013-08-28

**Authors:** Nadia Houri, Kuo-Cheng Huang, Josephine Nalbantoglu

**Affiliations:** Department of Neurology and Neurosurgery and Montreal Neurological Institute, McGill University, Montreal, Quebec, Canada; University Paris Sud, France

## Abstract

The Coxsackievirus and Adenovirus Receptor (CAR) is a cell adhesion molecule originally characterized as a virus receptor but subsequently shown to be involved in physiological processes such as neuronal and heart development, epithelial tight junction integrity, and tumour suppression. Proteolysis of cell adhesion molecules and a wide variety of other cell surface proteins serves as a mechanism for protein turnover and, in some cases, cell signaling. Metalloproteases such as A Disintegrin and Metalloprotease (ADAM) family members cleave cell surface receptors to release their substrates’ ectodomains, while the presenilin/ɣ-secretase complex mediates regulated intramembrane proteolysis (RIP), releasing intracellular domain fragments from the plasma membrane. In the case of some substrates such as Notch and amyloid precursor protein (APP), the released intracellular domains enter the nucleus to modulate gene expression. We report that CAR ectodomain is constitutively shed from glioma cells and developing neurons, and is also shed when cells are treated with the phorbol ester phorbol 12-myristate 13-acetate (PMA) and the calcium ionophore ionomycin. We identified ADAM10 as a sheddase of CAR using assays involving shRNA knockdown and rescue, overexpression of wild-type ADAM10 and inhibition of ADAM10 activity by addition of its prodomain. *In vitro* peptide cleavage, mass spectrometry and mutagenesis revealed the amino acids M224 to L227 of CAR as the site of ADAM10-mediated ectodomain cleavage. CAR also undergoes RIP by the presenilin/γ-secretase complex, and the intracellular domain of CAR enters the nucleus. Ectodomain shedding is a prerequisite for RIP of CAR. Thus, CAR belongs to the increasing list of cell surface molecules that undergo ectodomain shedding and that are substrates for ɣ-secretase-mediated RIP.

## Introduction

The Coxsackievirus and Adenovirus Receptor (CAR) is a cell adhesion molecule of the Immunoglobulin (Ig) superfamily [1,2]. As its name suggests, CAR is the attachment site of Coxsackie B viruses as well as some adenovirus serotypes [3–5]. Although much of the research on CAR has been within the context of adenovirus-mediated gene therapy, recent studies have described a wide range of physiological and pathophysiological roles.

CAR is highly conserved, especially in its C-terminus [2], and it is expressed in a variety of mammalian and non-mammalian species such as *D. rerio* and *X. laevis*. It is highly expressed in the developing nervous system, particularly in neuronal growth cones [6,7], and it mediates neurite extension by binding to the extracellular matrix protein fibronectin [8]. As a component of epithelial cell tight junctions, CAR participates in forming a barrier to paracellular flow of macromolecules, binding to tight junction proteins such as zonula occludens-1 (ZO-1) [9] and multi-PDZ domain protein-1 (MUPP-1) [10]. Although CAR expression is high in the developing heart and skeletal muscle, it is barely detectable by adulthood, becoming restricted to skeletal muscle neuromuscular junctions and cardiac intercalated discs [11,12]. CAR expression is critical for normal cardiac development, as its gene deletion in mice before embryonic day 11 results in cardiac abnormalities and embryonic lethality [13–15]. As well, CAR deletion in adulthood leads to multiple organ phenotypes including impairment of cardiac atrioventricular connection [16,17], atrophy of exocrine pancreas, enlarged intestines, and an increase in the number of thymocytes in the thymus [18].

In the immune system, CAR facilitates migration of neutrophils across epithelial cell tight junctions and endothelial cells during inflammatory episodes via its interaction with junctional adhesion molecule-like protein (JAML) [19,20]. Furthermore, epithelial CAR interacts with JAML in resident γδ T cells in the skin to promote effective γδ T cell response to changes in epithelial integrity [21]. Interestingly, CAR is downregulated in several cancers, and it inhibits the growth or invasion of some of these cancer cell lines when its expression is restored, such as in bladder cancer [22] and gliomas [23,24]. Therefore, CAR’s levels are tightly controlled and it functions in cell adhesion, migration and regulation of growth.

A wide variety of cell surface receptors are known to undergo proteolysis. One group of enzymes that cleave and release the ectodomains of cell surface proteins into the cellular environment is the A Disintegrin and Metalloprotease (ADAM) family of transmembrane and secreted metalloproteases. ADAMs act on diverse substrates including cytokines, cytokine receptors, cell adhesion molecules and growth factor receptors, with effects on a multitude of functions such as sperm maturation and sperm-egg adhesion, cell migration, axon guidance and cell fate determination in the nervous system [25]. Cell adhesion molecules that are shed by ADAMs include L1 [26], close homolog of L1 (CHL1) [27] and N-cadherin [28].

The γ-secretase complex participates in another type of proteolysis, regulated intramembrane proteolysis (RIP), which releases substrates’ intracellular domains into the cytosol. Intracellular domain (ICD) products of RIP are then degraded or participate in cell signaling [29]. Familial mutations in the *PSEN1* and *PSEN2* genes that encode presenilin (PS), the catalytic component of the γ-secretase complex, cause autosomal-dominant inherited Alzheimer’s disease [30]. Amyloid precursor protein (APP) is cleaved by PS/γ-secretase, and the resulting intracellular domain is rapidly degraded [29], although it has also been suggested to act as a transcriptional activator [31]. RIP of Notch produces an intracellular fragment that translocates to the nucleus and regulates transcription of Notch-responsive genes such as *HES* [32].

Given that many cell surface receptors, including cell adhesion molecules, undergo proteolysis, we wondered if that is the case for CAR. Here, we report that CAR is shed in a constitutive fashion as well as via activation of the protein kinase C (PKC) and calcium pathways. ADAM10 is a major sheddase of CAR ectodomain, and the site of ADAM10 cleavage on CAR is in the area of amino acids 224-227. CAR is also processed by γ-secretase, generating a 14 kDa intracellular domain fragment. Ectodomain shedding of CAR precedes its RIP. Finally, free CAR intracellular domain enters the nucleus as has been shown for a number of RIP substrates.

## Materials and Methods

### Ethics statement

This study was carried out in strict accordance with the Animal Care and Use Program Guidelines of McGill University. The protocol was approved by the Animal Care Committee of the Montreal Neurological Institute, McGill University (Permit Number: 2005-4971). Animals were sacrificed by CO_2_ and cervical dislocation, and all efforts were made to minimize suffering.

### Chemicals

Phorbol 12-myristate 13-acetate (PMA), O-phenanthroline, ionomycin and trichloroacetic acid (TCA) were from Sigma. GM6001, GM6001 negative control, Compound E (γ-Secretase Inhibitor XXI), recombinant human TIMP-1, TIMP-2 and TIMP-3, TAPI-1, Pepstatin, E-64, Leupeptin, DAPT, Gö 6983, and MG132 were from Calbiochem. Epoxomicin was from Biovision (Cedarlane). B-27 and N2 supplements were from Invitrogen. Purified prodomain of ADAM10 was a kind gift from Dr. Marcia Moss (Biozyme, Inc.).

### Antibodies

The production, purification and characterization of the rabbit polyclonal antibodies 2239 and 2240 raised against CAR N-terminal extracellular domain have been previously described [11]. The rabbit polyclonal antibody RP291 raised against the C-terminal intracellular domain of human CAR (46 kDa isoform) cross-reacts with the murine homolog mCAR1 [12,33], and was a kind gift from Dr. Kerstin Sollerbrant (Karolinska Institute and University Hospital, Stockholm, Sweden). Rabbit polyclonal antibody raised against ADAM10 was from AnaSpec, Inc. Mouse monoclonal antibody raised against the V5 tag was from Invitrogen. Horseradish peroxidase (HRP)-conjugated anti-glyceraldehyde -3-phosphate dehydrogenase (GAPDH) antibody was from Abcam. Goat or swine anti-mouse and anti-rabbit HRP-conjugated secondary antibodies were from Pierce and Dako. Goat anti-mouse Alexa Fluor 555 secondary antibody was from Molecular Probes.

### shRNA knockdown

Five different small hairpin ribonucleic acid (shRNA) sequences (TRC library) in a lentiviral vector (pLKO.1) targeting human ADAM10 and control shRNA targeting enhanced green fluorescent protein (eGFP) were purchased from Open Biosystems. Production of lentiviruses, infection and stable selection of cells with 2 μg/ml puromycin were performed according to the RNAi Consortium (TRC library) guidelines. Cell lines were maintained with 2 μg/ml puromycin. The level of knockdown in cells was initially assayed by real-time polymerase chain reaction (PCR) of reverse-transcribed RNA, normalizing over GAPDH. Only shRNA sequences that sufficiently knocked down *adam10* expression without affecting *adam17* levels were considered specific. Experiments were subsequently performed using the anti-ADAM10 hairpin sequence CCGGGCAGTATTACTTATGGGAATTCTCGAGAATTCCCATAAGTAATACTGCTTTTT (thereafter referred to as “6676”) or a second hairpin anti-ADAM10 shRNA, CCGGGCTGTGCAGATCATTCAGTATCTCGAGATACTGAATGATCTGCACAGCTTTTT (referred to as “6675”).

### Plasmids

Murine CAR (isoform 1) cloned in pcDNA3 plasmid has been previously described [11]. All point mutations or deletions were performed with the QuikChange II XL site-directed mutagenesis kit (Strategene), per manufacturer’s guidelines. Four mutants of CAR were generated using pcDNA3-mCAR1 as template: MLAA (in which amino acids M224 and L225 were mutated to alanine residues), RLAA (in which amino acids R226 and L227 were mutated to alanine residues), MLRLAAAA (in which M224, L225, R226 and L227 were mutated to alanine residues) and Δ221-232 (in which amino acids 221-232 were deleted).

The plasmid pcDNA 3.1/V5-His6x B (Invitrogen) was a kind gift from Dr. Alyson Fournier (McGill University). To generate the CAR-V5 construct (full-length murine CAR isoform 1 with a V5/His6x tag at the C-terminus), CAR insert in pcDNA3 plasmid was amplified with Phusion High Fidelity DNA polymerase (Finnzymes Inc.), including the Kozak sequence and excluding the stop codon. Blunt-end cloning of the PCR product was performed with EcoRV-digested pcDNA 3.1/V5-His. To obtain a construct of the intracellular domain of CAR with C-terminal V5-His6x tags (herein referred to as CAR ICD-V5), full-length CAR in pcDNA3.1 V5-His plasmid was used as template with the QuikChange II XL site-directed mutagenesis kit (Stratagene). Amino acids 2-260 inclusive were deleted from CAR-V5 to generate ICD-V5 using the QuikChange II XL site-directed mutagenesis kit (Stratagene).

Human ADAM10 cDNA in pCR4-TOPO plasmid (clone ID 8991969) was purchased from Open Biosystems and cloned into pcDNA3.1 plasmid using the NotI and PmeI sites. The shRNA-resistant mutant was generated from this construct by deleting nucleotides 2319-2325 using the QuikChange II XL site-directed mutagenesis kit (Strategene), per manufacturer’s guidelines.

The plasmid pWPI with IRES-GFP (Addgene ID # 12254) was a kind gift of Dr. Didier Trono. Full-length CAR with C-terminal V5/6xHis tag and stop codon was amplified by PCR using CAR-V5 (pcDNA3.1) plasmid as template. The PCR product was introduced into the pWPI vector at the PmeI site via blunt-end ligation.

All constructs were verified by sequencing at the Plateforme de séquençage et de génotypage des génomes, Centre de recherche du CHUL (Québec, Canada).

### Cell lines and culture conditions

The human embryonic kidney (HEK) 293, human glioma U251N and human glioma U87-MG cell lines were obtained from the American Type Culture Collection (ATCC) (Rockville, MD). The murine embryonic fibroblast (MEF) cell line knockout for PS 1 and 2 (herein referred to as “MEF PS 1/2 KO”) and wild-type MEF cell line were kind gifts from Dr. Bart de Strooper, K.U. Leuven, Belgium [34,35]. Cells were maintained in an incubator at 37°C with 5% CO_2_ using Dulbecco’s Modified Eagle Medium (DMEM) supplemented with 10% heat-inactivated fetal bovine serum (FBS) and antibiotic cocktail (100 units of penicillin/ml, 100 μg of streptomycin/ml).

The generation of U87-MG polyclonal cell populations over-expressing CAR via a retroviral vector has been previously described [11,24]. Briefly, retroviruses carrying the sequence for full-length murine CAR (isoform 1) were produced. Retroviruses carrying empty vector with the neomycin resistance gene were used as control. The supernatants from the producer cell lines were used for infection of U87-MG cells, which were then selected for 10 days with G418 (600 μg/ml). Clones were pooled together to generate bulk populations stably expressing CAR (U87 CAR) or control cells (U87 LNCX).

U87-MG, U251N and HEK 293 cells were transfected with wild-type or mutant CAR constructs using FuGENE 6 (Roche) or TransIT-2020 (Mirus) per manufacturers’ guidelines. Cells were selected for 7-10 days with 600 μg/ml G418 and then maintained with 200 μg/ml G418. Clones were pooled together during generation of stable cell lines in order to minimize clonal-specific effects. For some experiments that involved transient transfection of U87-MG and U251N cells with DNA, the aforementioned transfection reagents were also used.

MEF cells were infected with lentivirus for expression of V5-tagged CAR (in pWPI vector) per RNAi Consortium (TRC library) guidelines. Cells were lysed 3 days post-infection for analysis via SDS-PAGE and Western blot.

### Culture of murine embryonic hippocampal neurons

Cultures were prepared as previously described [7]. Timed pregnant mice (Charles River Laboratories) at 17 days gestation were sacrificed by CO_2_ and cervical dislocation, and embryos were sacrificed by decapitation. Hippocampus pairs were dissected from brains of embryos and collected into 4.5 ml of ice-chilled Hank’s Balanced Salt Solution (HBSS) supplemented with 1.0 mM of sodium pyruvate, penicillin (100 U/ml), and streptomycin sulfate (100 μg/ml). 0.5 ml of 2.5% trypsin was added (for a final concentration of 0.25% trypsin) and incubated at 37^°^C for 15 minutes, after which 0.5 ml of heat-inactivated FBS was added to the trypsinized samples. The samples were then triturated several times using a fire-polished glass Pasteur pipette. Dissociated individual cells were then separated from undissociated tissue debris by filtering the triturated sample through a cell strainer (70 μm) into a 15ml tube, and cells were pelleted by centrifugation for 3 minutes at 200 x *g* at room temperature. The cell pellet was then gently resuspended in Neurobasal medium supplemented with B-27 supplement, N2 supplement, 0.5 mM L-glutamine, 100 U/ml penicillin, and 100 μg/ml streptomycin sulfate.

### Preparation and collection of conditioned media

For PMA-induced, ionomycin-induced, or constitutive shedding of CAR, 2.5 x 105–5 x 10^5^ cells were seeded, respectively, per well of a 6-well plate. The following day, cells were washed and incubated in 1 ml of opti-MEM, either for 3-4 hours with PMA (10^-6^ M final concentration; dimethyl sulfoxide (DMSO) vehicle as control) or for 30 minutes with ionomycin (1.5 μM final concentration; DMSO vehicle as control). For constitutive shedding, cells were incubated for 16-24 hours in opti-MEM. At the end of the experiment, conditioned media were transferred into chilled microtubes. Cells were lysed with buffer containing 60 mM Tris-HCl pH 6.8, 4% sodium dodecyl sulfate (SDS), 10% glycerol and protease inhibitors (Roche Complete EDTA-free protease inhibitor cocktail), and heated at 95°C. The collected conditioned media were cleared of cell debris. The trichloroacetic acid (TCA) protein precipitation method was adapted with minor modifications from the protocol developed by the Bjorkman group (Howard Hughes Medical Institute, California Institute of Technology). Per 1 ml of conditioned media, 5 μg of bovine serum albumin (BSA) was added, followed by 250 μl of chilled 100% TCA (drop-wise). Samples were incubated overnight at 4°C and then pelleted at high speed in a microcentrifuge at 4°C, followed by two washes with ice-cold acetone. The acetone was evaporated by heating the samples briefly in a 60°C heating block. Protein pellets were solubilized in Laemmli/2-mercaptoethanol buffer.

### SDS-PAGE and Western blots

Protein concentrations of cell lysates were determined using the bicinchoninic acid (BCA) assay kit from Pierce Biotechnology, Inc. Samples were loaded on 13% SDS-PAGE gel, and electrophoresis was performed under reducing conditions. Precision Plus Protein Dual Color Standards (BioRad) and MagicMark XP (Invitrogen) were used to visualize band sizes on SDS-PAGE gel and Western blot, respectively. Proteins were transferred to polyvinylidene fluoride (PVDF) membranes (Immobilon P, Thermo Scientific) at 0.3 amps for 1 hour in a mini-transblot apparatus (BioRad). Transfer efficiency was verified with Ponceau staining, and membranes were then blocked in 10% (w/v) skim milk in Tris-buffered saline (TBS) containing 0.1% Tween-20 (TBS-T). Primary antibodies were diluted with 5% skim milk in TBS-T and applied to membranes with gentle shaking overnight at 4°C. After extensive washes with TBS-T, HRP-conjugated secondary antibody was applied with gentle shaking to membranes for 1 hour at room temperature. The membranes were extensively washed with TBS-T, and SuperSignal West Femto substrate (Pierce Biotechnology, Inc.) was applied per manufacturer’s guidelines. Chemiluminescence signal was detected with a cooled charge-coupled device (CCD) camera attached to an imaging capturing system (Gene-Gnome, Syngene) or on film (Denville HyBlot CL). Quantification of band intensities was performed using Syngene software or ImageJ. Western blots shown are representative of at least 3 independent experiments each.

### Calf intestinal phosphatase (CIP) treatments

Calf intestinal alkaline phosphatase (CIP) was purchased from New England Biolabs. U87 CAR cells were treated with 1 µM PMA (vs. DMSO vehicle) for 4 hours to stimulate CAR ectodomain shedding. Cells were lysed with lysis buffer and proteins were quantified using BCA protein assays as previously described. 20 µg of protein per sample were treated with 40 units of CIP (vs. H_2_O) for 1 hour at 37^°^C, followed by sample analysis via SDS-PAGE and Western blot.

### Reverse transcription of total RNA and real-time PCR

Total RNA was harvested from cells using the RNeasy Mini kit and QiaShredder (both from Qiagen), followed by reverse transcription with M-MLV reverse transcriptase (Invitrogen) according to the manufacturers’ guidelines. cDNA samples were diluted to fall within the standard curves, and samples were run in triplicate using an ABI Prism 7000 Sequence Detection System and SYBR Green PCR Core reagents (Applied Biosystems, Inc.) per manufacturer’s guidelines. Analyses were performed using the 7000 System software (Applied Biosystems, Inc.) and data were normalized over GAPDH transcript levels. Primers were designed using Primer Express 2.0 software (Applied Biosystems, Inc.) and the sequences used were as follows: human GAPDH-forward: 5’-CATCAATGACCCCTTCATTGAC -3’; human GAPDH-reverse: 5’-CGCCCCACTTGATTTTGGA-3’; human ADAM10-forward: 5’-GCGGCCCCGAGAGAGTTA-3’; human ADAM10-reverse: 5’-AGGAAGAACCAAGGCAAAAGC-3’; human ADAM17-forward: 5’-GGATACATGCTCTTAGAAAATTCACTATTG-3’; and human ADAM17-reverse: 5’-GCAACCTCAGCCTCTCCAAGT-3’. Different concentrations were tested for each pair of primers, and the optimal concentration for each pair was found to be 900 nM. Primers were synthesized by Alpha DNA (Montreal, Canada).

### In vitro enzymatic cleavage and mass spectrometry

A peptide with sequence VGSDQCMLRLDVVPPSNRAG representing amino acids 218-237 of murine CAR isoform 1 (mCAR1) was synthesized by GenScript, Inc. Recombinant human ADAM10 was purchased from R & D Systems, Inc. The buffer used for *in vitro* digestion contained 25 mM Tris, 2.5 μM ZnCl_2_ and 0.005% Brij-35 detergent at pH 9.0. 10 μg of peptide was digested with 2 μg recombinant enzyme in 10 μl final volume at 37°C for 4 or 16 hours. The following controls were also included: peptide only or recombinant ADAM10 only (16 hours digestion at 37°C for each), and peptide plus recombinant ADAM10 at 0 hours incubation (prepared on ice and stopped immediately). 20% trifluoroacetic acid (TFA) was used to stop each reaction with a final pH of 3. Samples were analyzed at the Genome Quebec Proteomics Platform (Montreal, Canada) via matrix-assisted laser desorption/ionization mass spectrometry (MALDI-MS) and tandem mass spectrometry (MS/MS).

### Cell surface biotinylation

Cells were seeded at a density of 10^6^ cells per 10 cm dish. The next day, cells were washed with phosphate-buffered saline (PBS) twice at 4°C and incubated with 7.5 mg of EZ-Link Sulfo NHS-LC-Biotin (Pierce Biotechnology) for 30 minutes at 4°C. The reaction was quenched by washing cells twice with 1 mM glycine, followed by two washes with PBS, all for 10 minutes each at 4°C. Cells were then harvested with modified RIPA buffer [20 mM HEPES pH8, 150 mM NaCl, 0.1% SDS, 1% Triton X-100, 1% sodium deoxycholate and protease inhibitor cocktail (Roche)], incubated on ice for 10 minutes, cleared by high speed centrifugation, and incubated with streptavidin beads (Pierce Biotechnology) for 2 hours at 4°C with gentle rotation. Streptavidin beads were washed 3 times with modified RIPA buffer, eluted with Laemmli/2-mercaptoethanol buffer, and boiled at 95°C for 5 minutes.

### Immunofluorescence

Cells were seeded on 22x22 mm glass square coverslips (grade 1.5) in 6-well plates and transfected the following day. Immunofluorescence experiments were performed 24-48 hours post-transfection. Cells were washed with PBS, fixed using 4% paraformaldehyde for 15 minutes at room temperature, and washed again with PBS. Cells were permeabilized using 0.1% Triton X-100 in PBS for 10 minutes at room temperature, followed by washing the samples with PBS. Samples were incubated for 1 hour at room temperature in blocking buffer (PBS with 10% goat serum). Primary antibody incubation using anti-V5 antibody at 1:500 dilution in blocking buffer was performed for 1 hour at room temperature, followed by a PBS wash and a 30 minute incubation at room temperature with goat anti-mouse Alexa Fluor 555 secondary antibody (1:1000 dilution in blocking buffer). Nuclei were stained for 5 minutes using DRAQ5 (Cell Signaling Technology) diluted 1:3000 in PBS. Coverslips were mounted on slides using ProLong Gold Antifade Reagent (Molecular Probes).

### Confocal microscopy

Images of samples were acquired using a Zeiss LSM 510 Meta laser scanning confocal microscope with a Plan-Apochromat 63x/1.4 oil DIC objective (Cell Imaging and Analysis Network, McGill University). Multitrack mode was used with dual excitation (633 nm for DRAQ5 and 543 nm for Alexa Fluor 555) and emission (LP 650 nm for DRAQ5 and BP 560-615 nm for Alexa Fluor 555) filter sets.

### Statistical analyses

All values reported are expressed as mean ± standard error of the mean (SEM). Statistical significance was assessed by Student’s t-test or by one-way analysis of variance (ANOVA) as stated in the figure legends using GraphPad Prism software version 3.0. Statistical significance was defined as p < 0.05.

## Results

### CAR sheds its ectodomain

**Figure 1:**
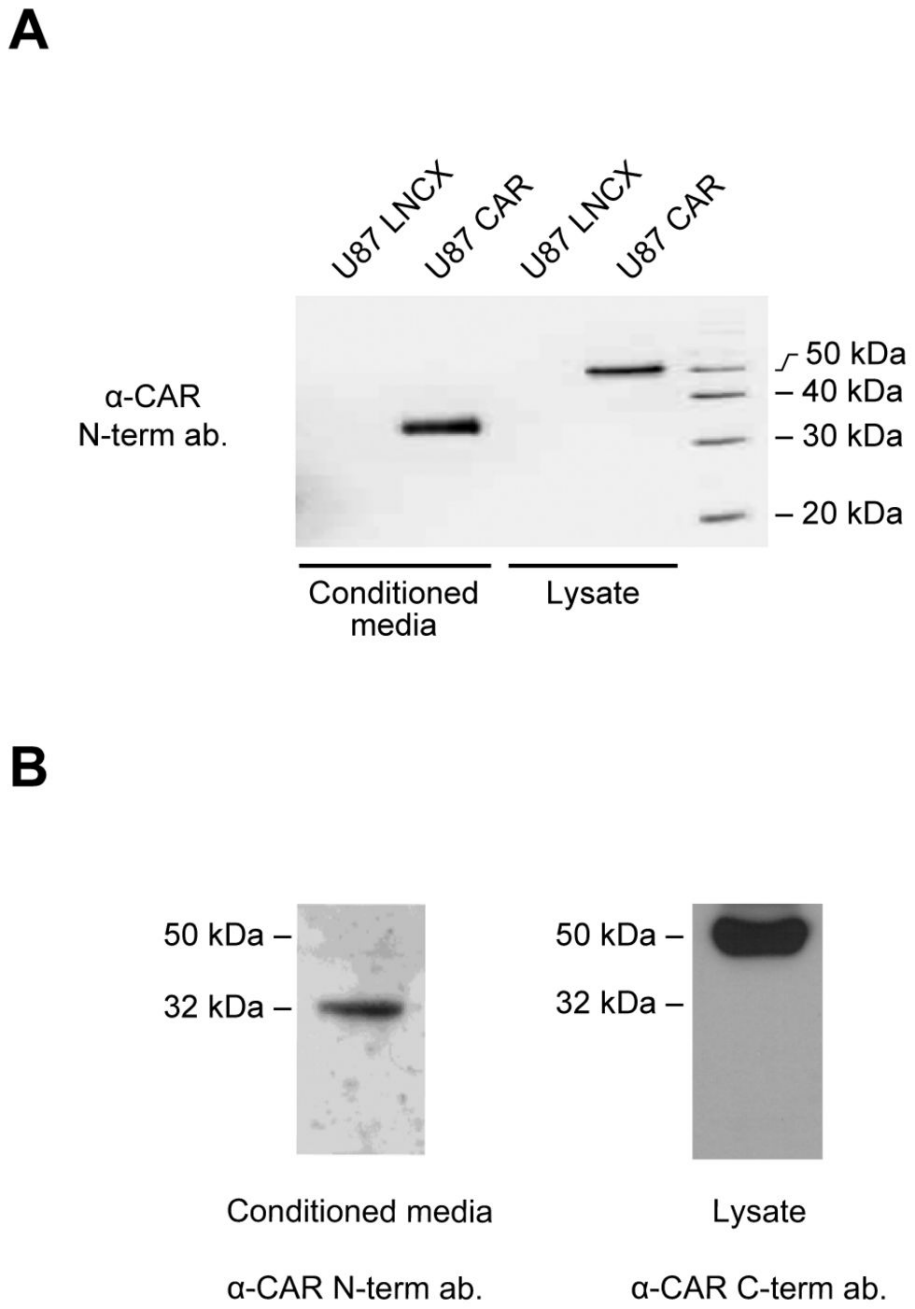
An extracellular fragment of CAR is detected from conditioned media of human glioma U87-MG cells and mouse embryonic hippocampal neurons. (A) A Western blot of conditioned media and cell lysates from U87 LNCX (control) and U87 CAR cells using the anti-CAR N-terminus antibody 2240. The shed extracellular fragment detected from conditioned media migrates at approximately 32 kDa, while full-length CAR detected from cell lysates migrates at approximately 50 kDa. (B) A Western blot of conditioned media from embryonic hippocampal neurons (3 days *in vitro*), using the anti-CAR N-terminus antibody 2240, revealed the presence of a 32 kDa fragment, similarly to the U87-MG CAR-expressing cell line. Also shown is a Western blot of full-length CAR detected from neuronal lysate using the anti-CAR C-terminus antibody RP291.

To investigate the possibility that CAR ectodomain (ECD) is released from cells into the extracellular environment, we collected conditioned media from a human glioma cell line U87-MG stably expressing CAR (U87 CAR), and performed Western blot analyses using rabbit polyclonal antibodies raised against the extracellular domain [11]. CAR (approximately 50 kDa in molecular weight) was detected from lysates of U87 CAR cells (Figure 1A), while no CAR was detected from the control, vector-infected cell line (U87 LNCX). A fragment of CAR with a lower molecular weight (approximately 32 kDa) was detected from conditioned media of U87 CAR cells; the molecular weight of this fragment corresponds to the expected size of CAR ECD (Figure 1A). Furthermore, this 32 kDa fragment was not recognized by RP291 antibody raised against CAR’s intracellular domain [12,33] (Figure S1), confirming that this shed fragment is indeed CAR ECD. Shedding was not limited to exogenous expression in stable cell lines, as a fragment of the same size was also detected from conditioned media of murine embryonic hippocampal neurons which express CAR endogenously (Figure 1B) [6,7].

**Figure 2:**
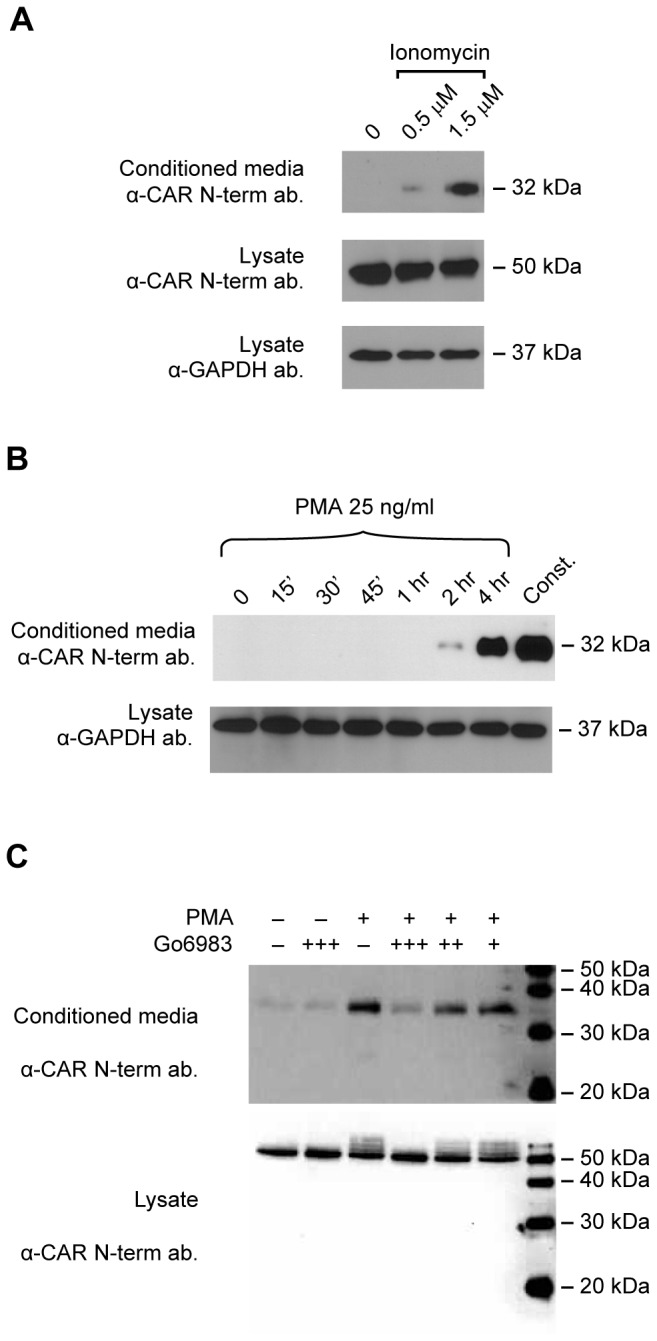
CAR shedding is stimulated by the calcium ionophore ionomycin and the phorbol ester PMA. (A) U87 CAR cells were treated with the calcium ionophore ionomycin at the indicated concentrations for 30 minutes. Ionomycin treatment stimulated the ECD shedding of CAR. (B) PMA treatment (25 ng/ml) of U87 CAR cells did not trigger CAR ECD shedding within one hour. At this concentration, 4 hours of PMA treatment led to robust CAR ECD shedding, but remained lower than constitutive shedding over 16 hours. Volumes of conditioned media loaded on SDS-PAGE were adjusted according to lysate protein concentrations. (C) The protein kinase C (PKC) inhibitor Gö 6983 decreased PMA-stimulated shedding of CAR ECD into conditioned media in a dose-dependent manner. PMA was used at a final concentration of 1 μM, and Gö 6983 at the following concentrations: + = 1 nM; ++ = 10 nM, and +++ = 100 nM. For the Western blots shown in these panels, the anti-CAR N-terminus antibodies 2239 or 2240 were used.

ECD shedding of cell surface proteins can be controlled by cell signaling pathways, with regulated stimulation of ECD shedding occurring within a relatively short period of time compared to constitutive shedding. For example, the calcium ionophore ionomycin can induce rapid cell surface protein shedding [36] through stimulation of ADAM10 [37,38]. As seen in Figure 2A, treatment of U87 CAR cells with ionomycin for 30 minutes upregulated CAR ECD shedding. Phorbol esters such as PMA activate the PKC pathway [39] and also induce shedding of cell surface proteins [40]. Shedding of substrates via treatment with low concentrations of PMA for 1 hour or less is generally mediated by ADAM17 [37,41]. In the case of U87 CAR cells treated with 25 ng/ml of PMA, substantial levels of CAR ECD were detected from conditioned media only after 2 hours of treatment, suggesting that ADAM17 may not be involved in CAR ECD shedding (Figure 2B). PMA-induced shedding of CAR ECD was inhibited by the PKC inhibitor Gö 6983 in a dose-dependent manner (Figure 2C). Thus, CAR shedding is regulated by signaling pathways.

During the course of these studies, we noticed that PMA treatment was accompanied by the appearance of higher molecular weight bands of CAR in cell lysates (Figure 2C), a phenomenon that was not observed with constitutive or ionomycin-mediated ECD shedding. We hypothesized that PMA treatment of U87 CAR cells leads to a post-translational modification of the full-length receptor in the form of phosphorylation. Indeed, treatment of lysates with calf intestinal phosphatase (CIP) abolished the appearance of these bands (Figure S2). This modification of CAR was dependent on PKC activity (Figure 2C).

**Figure 3:**
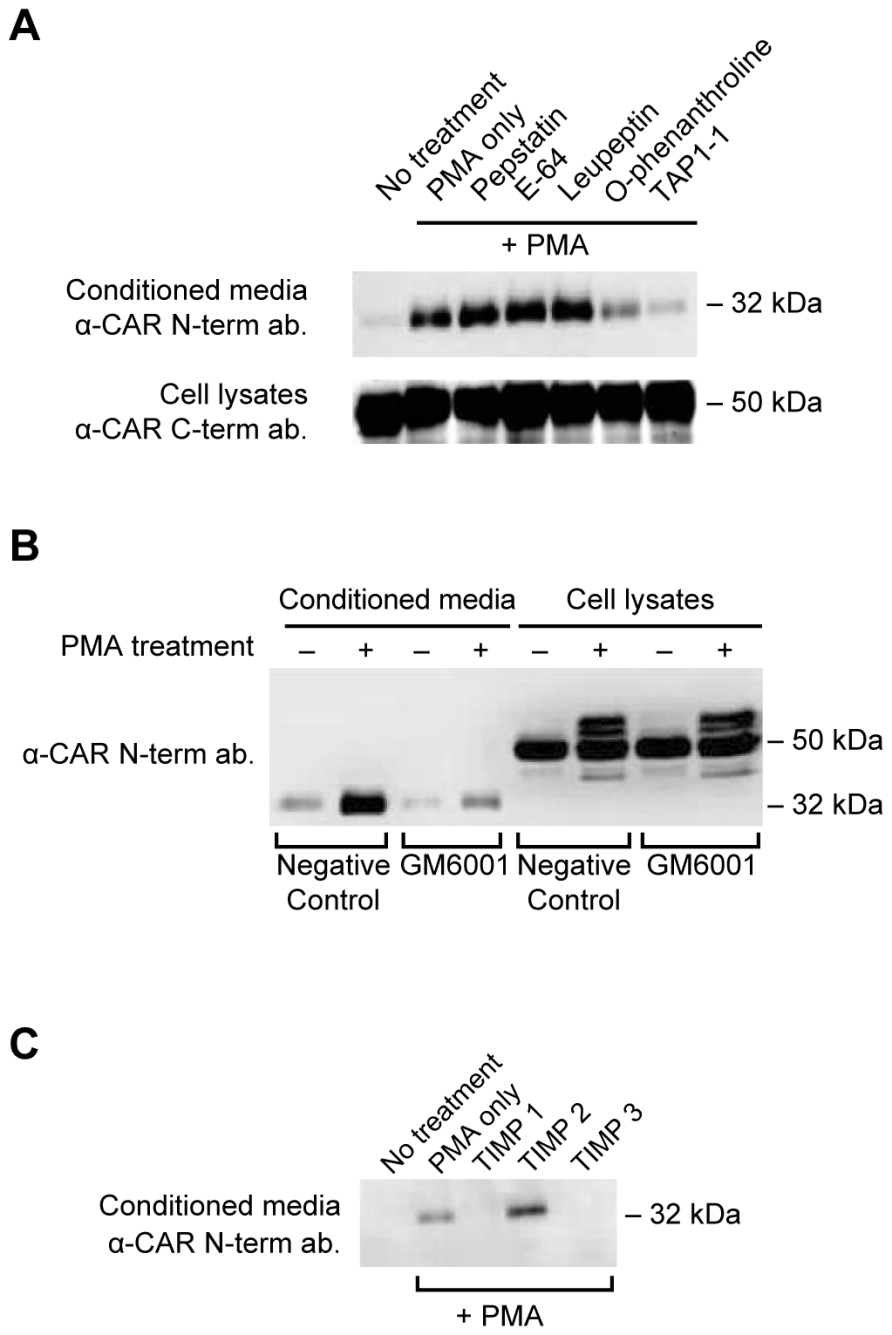
CAR shedding is mediated by metalloproteases. (A) U87 CAR cells plated on poly-L-lysine coated plates were pre-incubated for 45 minutes with a variety of protease inhibitors (10 µM pepstatin A, 10 µM leupeptin, 10 µM E64, 250 µM O-phenanthroline, 25 µM TAPI-1) followed by 3 hours of treatment with 1 µM of PMA. None of the treatments were toxic to the cells under these conditions and concentrations of inhibitors. CAR ECD released into conditioned media was detected via Western blot using anti-CAR N-term. antibody 2240. The broad-spectrum metalloprotease inhibitors TAPI-1 and O-phenathroline decreased PMA-stimulated CAR ECD shedding, while the aspartyl protease inhibitor pepstatin, the cysteine protease inhibitor E64, and the cysteine/serine protease inhibitor leupeptin had no effect. Also shown are Western blots of full-length CAR from the corresponding cell lysates (anti-CAR C-term. antibody RP291). (B) U87 CAR cells were treated with PMA (1 µM) or DMSO vehicle, in the presence of 25 µM of the broad spectrum metalloprotease inhibitor GM6001 or its negative control. GM6001, but not its negative control, inhibited PMA-stimulated shedding of CAR ECD. (C) U87 CAR cells were incubated for 3 hours with 1 µM PMA along with 10 µg/ml of TIMPs 1, 2 or 3. TIMP1 and TIMP3, but not TIMP2, decreased PMA-mediated ECD shedding of CAR, suggesting that ADAM10 may be a sheddase. For the Western blots shown in these panels, the anti-CAR N-terminus antibodies 2239 or 2240 were used.

To determine which protease family mediates the ECD shedding of CAR, various protease inhibitors were used in conjunction with PMA treatment of U87 CAR cells. The broad-spectrum metalloprotease inhibitors O-phenathroline and TAPI-1 inhibited PMA-stimulated CAR ECD shedding, but the aspartyl protease inhibitor pepstatin, the cysteine protease inhibitor E64, and the cysteine/serine protease inhibitor leupeptin did not (Figure 3A). GM6001, another broad-spectrum metalloprotease inhibitor, inhibited PMA-stimulated CAR ECD shedding (Figure 3B) as well as constitutive shedding (data not shown). Thus, metalloproteases, and not other classes of proteases, are required for constitutive and PMA-induced ECD shedding of CAR.

To further confirm the involvement of metalloproteases, U87 CAR cells were treated with physiological tissue inhibitors of metalloproteases (TIMPs), which inhibit ADAMs and matrix metalloproteinases (MMPs) [42]. While TIMP1 and TIMP3 completely blocked PMA-stimulated shedding of CAR, TIMP2 did not have an effect (Figure 3C). TIMP1 inhibits ADAM10, and TIMP3 inhibits ADAM10, ADAM12, ADAM17, ADAM28 and ADAM33 [25]. Given that ionomycin treatment stimulated CAR ECD shedding (Figure 2A), that ADAM10 in general mediates ionomycin-induced shedding of substrates [37,38], and that ADAM17 is likely not involved in CAR ECD shedding (Figure 2B), we chose to further investigate ADAM10 as a candidate sheddase of CAR.

### ADAM10 mediates shedding of CAR ECD

**Figure 4:**
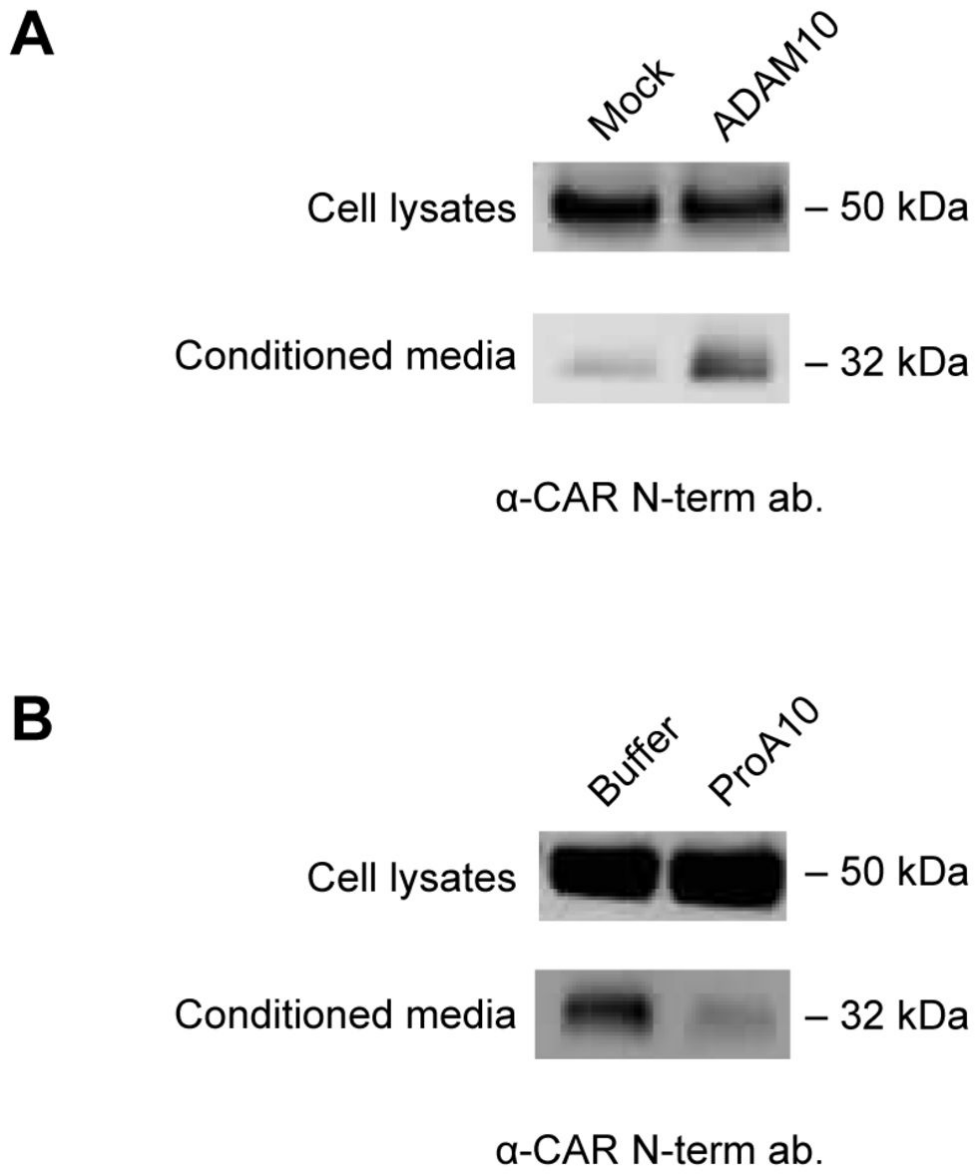
ADAM10 is involved in CAR ECD shedding. (A) U251N cells stably expressing CAR were transfected with empty plasmid (mock) or ADAM10 plasmid. 24 hours after transfection, cells were washed and incubated in opti-MEM for 24 hours, and conditioned media and cell lysates were analyzed by Western blots using anti-CAR N-terminus antibody (2239). Overexpression of ADAM10 increased constitutive CAR ECD shedding. (B) U87 CAR cells were treated with 10 μM purified ADAM10 prodomain (versus an equivalent volume of buffer as a control), and conditioned media and cell lysates were collected as previously described. A Western blot for CAR extracellular domain (2240 antibody) shows that the prodomain of ADAM10, which inhibits ADAM10 activity, decreased CAR ECD shedding.

Transient overexpression of wild-type ADAM10 in human glioma U251N cells stably expressing CAR (U251N CAR) increased shedding of CAR ECD (Figure 4A). Purified recombinant prodomain of ADAM10 inhibits ADAM10 activity when added to cell culture media [43]. Addition of ADAM10 prodomain markedly decreased constitutive shedding of CAR (Figure 4B). These results suggest that the metalloprotease ADAM10 is involved in CAR ECD shedding.

**Figure 5:**
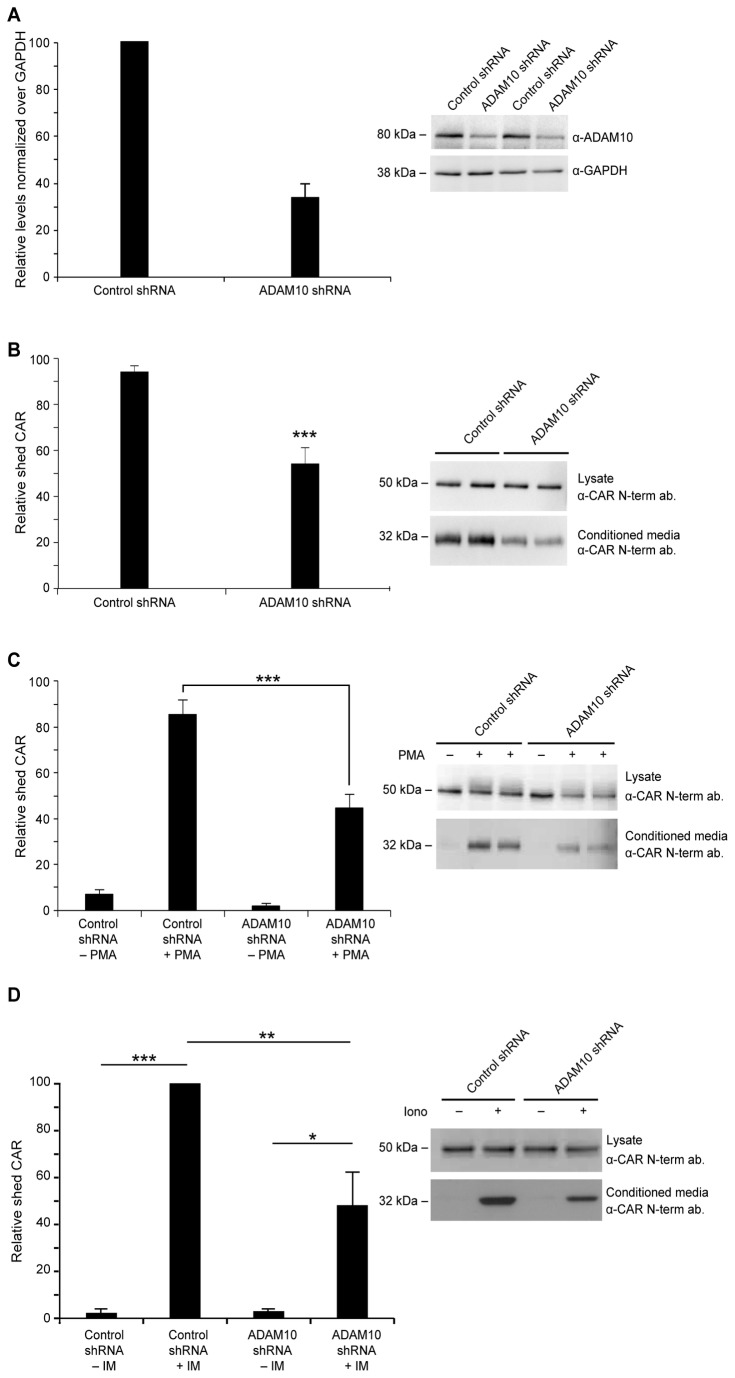
shRNA knockdown of ADAM10 decreases constitutive, PMA-mediated and ionomycin-mediated CAR ECD shedding. (A) A Western blot for ADAM10 using cell lysates of U87 CAR cells containing either control shRNA or ADAM10 (#6676) shRNA, in biological duplicates. Equal amounts of proteins were loaded on SDS-PAGE gel. Anti-GAPDH antibody was used as a loading control. Quantification of mean ADAM10 band intensities normalized over GAPDH revealed a decrease of approximately 60% with anti-ADAM10 shRNA compared to control shRNA. (B) A constitutive shedding experiment was performed using U87 CAR stable cell lines containing either control shRNA or anti-ADAM10 shRNA (#6676). Conditioned media and cell lysates were collected after 24 hours of incubation of cells in opti-MEM, and Western blotting was done using the anti-CAR N-terminus antibody 2239. With anti-ADAM10 shRNA, there was a significant decrease (40%) in the levels of shed CAR. Results from 4 independent experiments performed in duplicates were quantified (unpaired t-test; p=0.0004 (***)). (C) U87 CAR cells containing either control shRNA or ADAM10 shRNA (#6676) were treated with 1 µM PMA. Conditioned media and cell lysates were collected after 3 hours, and Western blots were performed using the anti-CAR N-terminus antibody 2239. With shRNA knockdown of ADAM10, there was a significant decrease of 41% in levels of shed CAR compared to control shRNA. Results from 3 independent experiments performed in duplicates were quantified (one-way ANOVA with Tukey’s multiple comparison test; *** = p < 0.001). (D) U87 CAR cells containing either control shRNA or ADAM10 shRNA (#6675) were treated with 1.5 µM ionomycin (vs. DMSO vehicle). Conditioned media and cell lysates were collected after 30 minutes of treatment, and Western blots were performed using the anti-CAR N-terminus antibody 2240. With shRNA knockdown of ADAM10, there was a significant decrease of 52% in levels of shed CAR with ionomycin treatment compared to control shRNA. Results from 3 independent experiments (n=3 per group) were quantified (one-way ANOVA with Bonferroni’s multiple comparison test; * = p < 0.05, ** = p < 0.01, *** = p < 0.001).

To further confirm ADAM10’s role in CAR shedding, we used shRNA to knock down ADAM10 expression in U87 CAR cells. Stable cell lines were generated for anti-eGFP shRNA (used as control) or anti-ADAM10 shRNA (sequences #6675 or #6676). The shRNA sequences targeting ADAM10 successfully knocked down ADAM10 mRNA levels (Figure S3) and, subsequently, ADAM10 protein levels (Figure 5A). The shRNA stable cell lines were then used to study constitutive and regulated CAR ECD shedding. Knockdown of ADAM10 in U87 CAR cells significantly decreased levels of constitutively shed CAR by 40% (Figure 5B). Similar results were obtained with the ADAM10 shRNA sequence #6675 (data not shown). PMA-mediated shedding of CAR also significantly decreased (by 41%) with knockdown of ADAM10 (Figure 5C), although it should be noted that the concentration and length of time used for the PMA treatments is considered to be chronic with a wide range of pleiotropic cellular effects; thus at these conditions, PMA activation of ADAM10 may be non-specific. Ionomycin-induced shedding of CAR ECD (following treatment of U87 CAR cells at 1.5 µM for 30 minutes) was also found to be ADAM10-dependent (Figure 5D). These data confirm that ADAM10 mediates constitutive, ionomycin-stimulated, and chronic PMA-stimulated ECD shedding of CAR.

**Figure 6:**
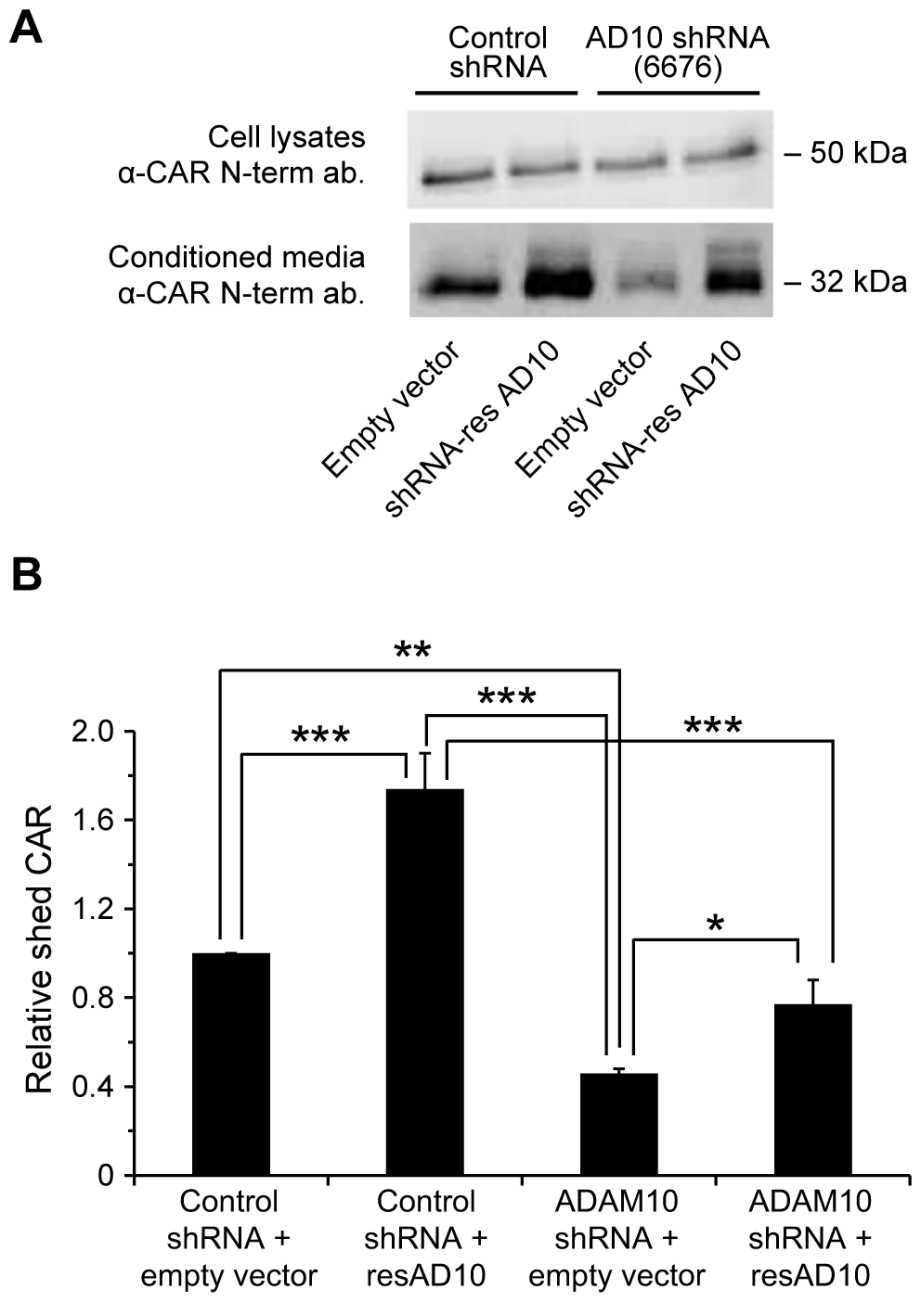
Partial rescue of shRNA-mediated loss of CAR shedding using an shRNA-resistant mutant of ADAM10. (A) shRNA stable cell lines of U251N CAR cells were transfected with either empty plasmid (mock) or an shRNA-resistant construct of ADAM10. 18-24 hours post-transfection, cells were washed and incubated in opti-MEM. Conditioned media and cell lysates were collected 24 hours later and analyzed by Western blot for CAR extracellular domain (anti-CAR antibody 2239). The shRNA-resistant ADAM10 mutant partially rescued CAR shedding in the ADAM10 (6676) shRNA cell line, compared to transfecting this cell line with empty plasmid (third and fourth Western blot bands from the left). (B) The band intensities of shed CAR detected by Western blot were quantified from 4 independent experiments (One-way ANOVA with Newman-Keuls multiple comparison test; * = p < 0.05, ** = p < 0.01, *** = p < 0.001).

For additional validation of ADAM10’s role in CAR ECD shedding, we generated a mutant ADAM10 construct resistant to shRNA #6676 by deleting nucleotides 2319-2325 (the target sequence of shRNA #6676, lying outside the coding sequence of human ADAM10). U251N CAR cells stably expressing control shRNA or ADAM10 shRNA were transiently transfected with either the shRNA-resistant ADAM10 plasmid or empty plasmid. The shRNA-resistant ADAM10 mutant partially rescued the ADAM10 shRNA-mediated decrease in CAR shedding (Figure 6). Thus, these results provide further evidence for ADAM10’s role in CAR ECD shedding.

### Mapping the site of cleavage within CAR’ s ECD

As ADAMs do not have consensus cleavage sites, *in vitro* experiments were performed using recombinant human ADAM10 to map the putative cleavage site(s) within CAR’s ECD. A peptide consisting of the 20 amino acid residues upstream of the transmembrane domain of murine CAR (isoform 1) having the sequence VGSDQCMLRLDVVPPSNRAG was digested *in vitro* with recombinant ADAM10 at 37°C for 4 or 16 hours. The following controls were also included: peptide only or enzyme only (16 hours of incubation at 37°C each) and peptide with enzyme for 0 hours. Samples were analyzed by MALDI-MS, and unique peaks at 1008 m/z and 1393 m/z were found that did not appear in the three control samples (Figure S4). MS/MS fragmentation was performed to deduce the amino acid identity of the fragments. The 1008 m/z peak corresponded to the peptide fragment VGSDQCMLR, whereas the 1393 m/z peak corresponded to the peptide fragment LRLDVVPPSNRAG.

**Figure 7:**
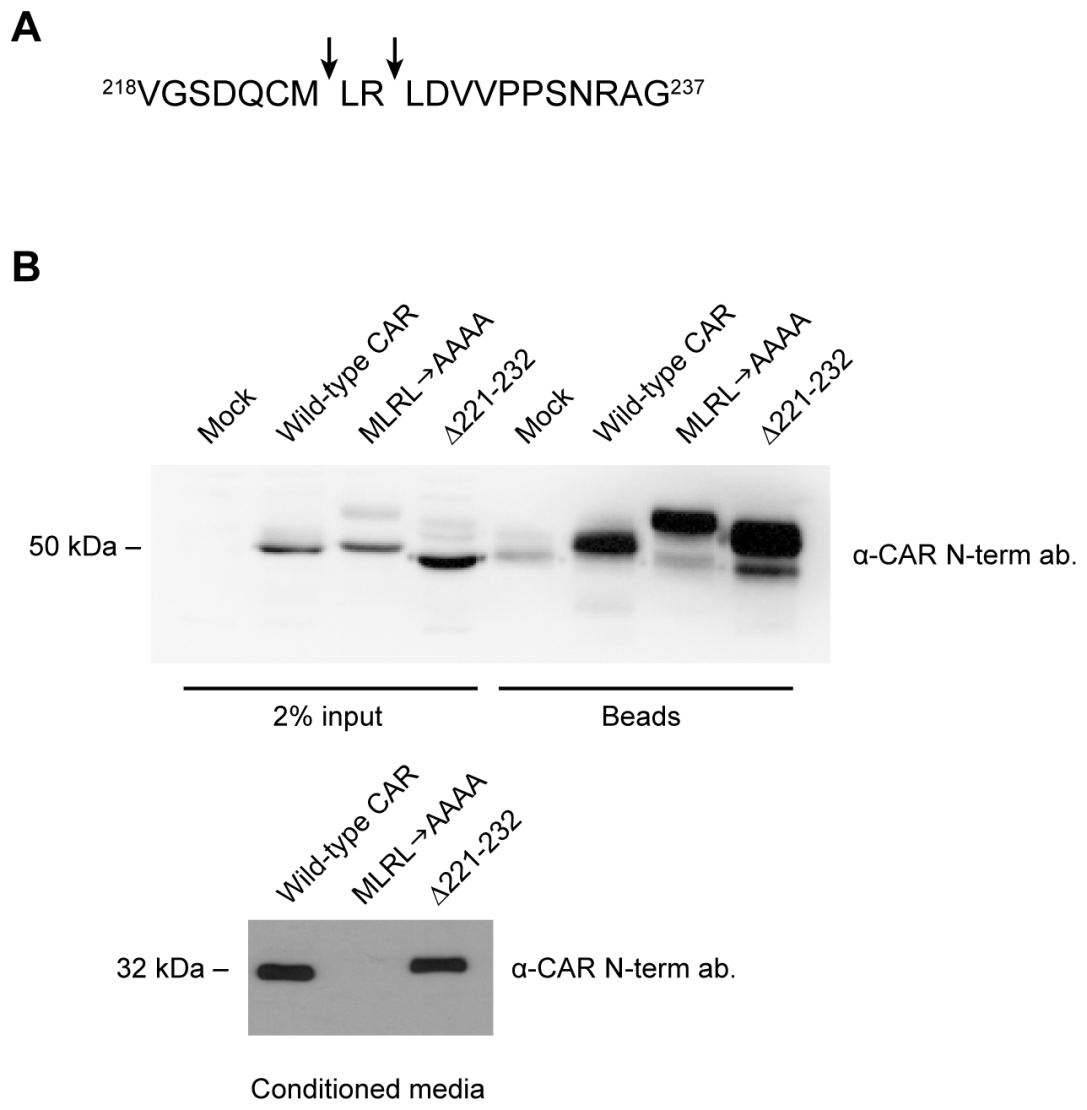
Confirmation of the area of ECD cleavage on CAR in HEK 293 cells. (A) A schematic showing putative ADAM10 cleavage sites on CAR’s extracellular domain (arrows), as obtained from *in vitro* peptide digestion and mass spectrometry (Figure S4). (B) HEK 293 stable cell populations were generated to express wild-type CAR, the mutant MLRLAAAA or the mutant Δ221-232. The MLRLAAAA mutant did not shed into conditioned media of HEK 293 cells, while the Δ221-232 mutant shed its ECD similarly to wild-type CAR. Cell surface biotinylation experiments revealed that wild-type CAR and the two mutants are expressed similarly on the cell surface. Note that the low level of endogenous CAR in HEK 293 was detected after enrichment of cell surface biotinylated proteins (mock lane). Western blotting was performed using the anti-CAR N-terminus antibody 2240.

Figure 7A illustrates the putative cleavage sites as determined from the mass spectrometry results: between M224 and L225, and between R226 and L227. To verify the cleavage sites, we generated CAR constructs in which these pairs of amino acids were mutated to alanines and the mutants named ML AA and RLAA, respectively. A third mutant (Δ221-232) was generated in which 12 amino acids (amino acids 221-232 inclusive), comprising most of the peptide sequence used for the *in vitro* digestion experiments, were deleted. Wild-type CAR and the mutants MLAA, RLAA, and Δ221-232 were stably expressed in U251N cells.

The MLAA and RLAA point mutants displayed abrogated ECD shedding, although not completely or consistently over subsequent passages of the stable cell lines (Figure S5A). Therefore, a third mutant of CAR was generated in which amino acids M224 to L227, inclusive, were changed to alanine residues (MLRLAAAA). This mutant was found to be completely defective in ECD shedding (Figure S5B), and this effect was consistent over further passages of these stable cell lines. Similar results were obtained in U87-MG cells (data not shown). Although we expected the deletion mutant Δ221-232 not to shed since it lacked most of the amino acid sequence used in the *in vitro* digestion experiments, this mutant’s ECD shed robustly, generating a slightly larger fragment than that of wild-type CAR (Figure S5A and Figure S5B). As the deleted region contains one of the cysteines that participate in a disulfide bridge [44], the structure of Δ221-232 is presumably disrupted, possibly exposing additional cleavage sites.

However, these mutations may alter the trafficking of CAR in glioma cells as cell surface biotinylation experiments revealed that the mutants were expressed at very low levels on the cell surface compared to wild-type CAR (Figure S5C and Figure S5D). On the other hand, when we performed similar studies on HEK 293 cells, which express CAR endogenously, stable transfection of wild-type CAR, MLRLAAAA and Δ221-232 revealed that the two mutants are expressed on the cell surface similarly to wild-type CAR and that the MLRLAAAA is indeed defective in ECD shedding (Figure 7B). Taken together, these results indicate that amino acids M224-L227 are important for cleavage of CAR ECD.

### CAR undergoes RIP mediated by the γ-secretase complex

**Figure 8:**
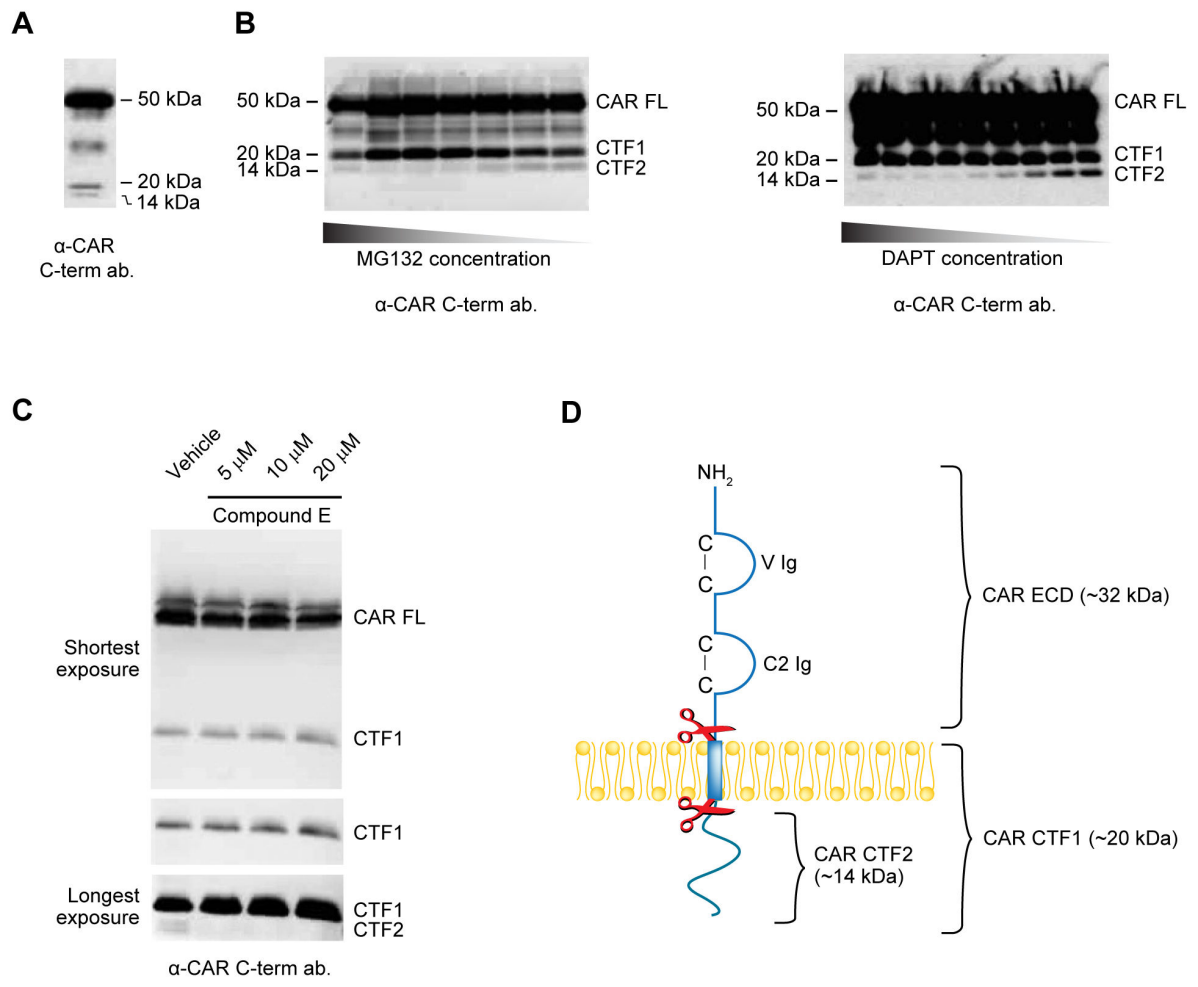
CAR undergoes RIP by the γ-secretase complex. (A) A Western blot for CAR’s intracellular domain (antibody RP291) shows lower molecular weight fragments in U87 CAR lysates at approximately 20 kDa and 14 kDa (CTF1 and CTF2, respectively). Full-length CAR (CAR FL) migrates at 50 kDa. (B) U87 CAR cells were treated with the γ-secretase inhibitors MG132 or DAPT for 16 hours. Equal amounts of proteins from cell lysates were analyzed by Western blot using the anti-CAR intracellular domain antibody RP291. Drug treatments resulted in accumulation of CAR CTF1 (20 kDa) and a decrease in CAR CTF2 (14 kDa) levels in a dose-dependent manner. (C) Overnight treatment of U87 CAR cells with Compound E, another inhibitor of the γ-secretase complex, at the indicated concentrations also diminished RIP of CAR. Equal quantities of proteins from cell lysates were analyzed by SDS-PAGE and Western blot using the anti-CAR intracellular domain antibody RP291. With increasing concentrations of Compound E, there was a corresponding accumulation of a 20 kDa fragment (CAR CTF1) and a decrease in a 14 kDa fragment (CAR CTF2). 3 different exposure times of the Western blot are shown. (D) A model of CAR proteolysis, with molecular weights of the resulting fragments indicated. Cleavage of CAR by ADAM10 (represented by the top pair of scissors) releases a 32 kDa fragment (CAR ECD) into the extracellular environment. The remaining 20 kDa fragment (CTF1) is processed by the γ-secretase complex (represented by the lower pair of scissors), generating a 14 kDa fragment (CTF2).

Often, cell surface proteins that undergo ECD shedding are also cleaved by the γ-secretase complex, resulting in the release of their intracellular domains into the cytosol. To investigate if smaller fragments are generated from CAR, the anti-CAR intracellular domain antibody RP291 was used in Western blots of U87 CAR cell lysates. Besides the full-length form of CAR (CAR FL), two C-terminal fragments (CTF1 and CTF2) of approximately 20 kDa and 14 kDa, respectively, were detected from cell lysates (Figure 8A).

We hypothesized that CTF1 is the remaining part of CAR after its ECD is shed, and that CTF2 is the product of the subsequent RIP of CTF1. If so, then inhibition of regulated intramembrane proteases such as the γ-secretase complex should lead to a decrease in CTF2 levels. Indeed, we found that treatment with the γ-secretase inhibitors MG132 and DAPT decreased CTF2 in a dose-dependent manner, and that this phenomenon was accompanied by an accumulation of CTF1 levels (Figure 8B). Similar results were obtained with a third γ-secretase inhibitor, Compound E (Figure 8C). The products of proteolytic cleavage of CAR and their molecular weights are illustrated in Figure 8D.

**Figure 9:**
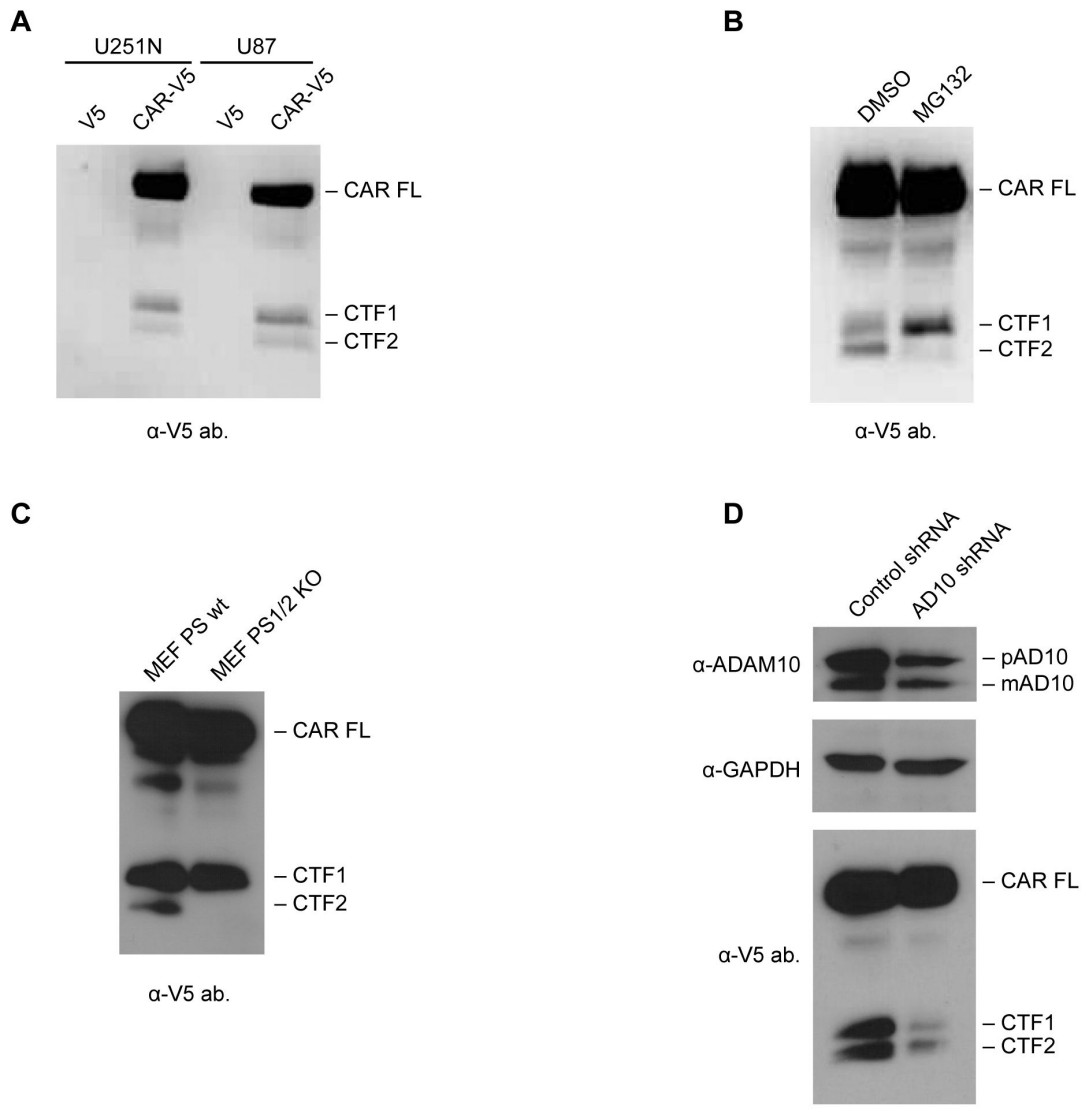
Generation of CAR CTF1 precedes CTF2 production. (A) Stable cell lines of U87-MG and U251N expressing either the V5 tag alone (mock) or CAR with a C-terminal V5 tag were generated. Equal amounts of cell lysates were analyzed by SDS-PAGE and Western blot using a mouse monoclonal antibody raised against the V5 tag. Full-length CAR, CTF1 and CTF2 were detected similarly to lysates of U87 CAR cells probed with anti-CAR C-term. antibody RP291 (Figure 8A). (B) U251N V5 and U251N CAR-V5 cells were treated overnight with MG132 (25 µM) or DMSO vehicle control. Equal amounts of proteins from cell lysates were used for anti-V5 Western blots. In the case of the MG132-treated cells, CTF1 levels accumulated while CTF2 nearly disappeared, similar to previous experiments with the U87 CAR cell line (Figure 8B). (C) MEF wild-type (MEF WT) or PS 1- and 2-knockout MEF cells (MEF PS1/2 KO) were infected with lentivirus to express full-length CAR with a C-terminal V5 tag. Cells were lysed 3 days post-infection and lysates were analyzed by Western blot using antibody raised against the V5 tag. MEF WT cells, but not MEF PS1/2 KO cells, contained CAR CTF2, indicating that presenilin is required for generation of the 14 kDa CTF2 fragment of CAR. (D) Verification of knockdown in ADAM10 expression in U87 CAR-V5 cells using ADAM10 shRNA (#6675); shown are anti-ADAM10 and anti-GAPDH Western blots. Lysates were also analyzed by Western blot using antibody raised against the V5 tag. The ADAM10 shRNA stable cell line had a decreased CAR CTF1 level, as expected. CAR CTF2 levels also decreased, indicating that shedding is a prerequisite for RIP of CAR.

The results in Figure 8 were additionally verified by stably expressing a CAR construct with a C-terminal V5 tag (CAR-V5) in U87-MG and U251N cells. Western blots of lysates from these cell lines using an anti-V5 antibody revealed the presence of CAR FL as well as two lower molecular weight species, CAR CTF1 (20 kDa) and CAR CTF2 (14 kDa) (Figure 9A), similar to fragments of intracellular CAR detected with the RP291 antibody (Figure 8A). When CAR-V5-expressing U251N cells were treated with MG132 to inhibit γ-secretase, CTF1 levels accumulated while CTF2 levels decreased (Figure 9B), similar to results obtained using the anti-CAR intracellular domain antibody RP291 (Figure 8B). Thus, in both U87-MG and U251N glioma cells, V5-tagged CAR undergoes RIP like untagged CAR.

As PS is the catalytic subunit of the γ-secretase complex [45], we hypothesized that lack of PS expression would result in absence of the 14 kDa CAR CTF2 fragment. MEF wild-type (WT) and MEF PS 1 and 2 knockout (PS 1/2 KO) cells were infected with a lentiviral vector expressing CAR with a C-terminal V5 tag. Lysates were analyzed by Western blot using antibody raised against V5. Although CAR CTF2 was readily detected in MEF WT cells, it was absent in cells null for PS 1 and 2 (Figure 9C). Thus, the PS/γ-secretase complex is required for RIP of CAR, resulting in generation of a 14 kDa fragment, CTF2.

To determine whether or not RIP of CAR is contingent upon its ECD shedding, U87 CAR-V5 cells were treated for 4 hours with 25 µM of the metalloprotease inhibitor GM6001 or its negative control. Inhibition of CAR ECD shedding was accompanied by a decrease in levels of both CTF1 and CTF2 (Figure S6). For further confirmation, ADAM10 was knocked down in U87 CAR-V5 cells (ADAM10 shRNA #6675), with control shRNA for comparison. The ADAM10 shRNA cell line had reduced amounts of the 20 kDa CTF1 fragment, as expected (Figure 9D), since a decrease in ECD shedding is expected to be accompanied by lower CTF1 levels. Importantly, levels of the 14 kDa CAR CTF2 also decreased. These data indicate that RIP of CAR is dependent on ECD shedding of the full-length receptor.

### CAR intracellular domain enters the nucleus

**Figure 10:**
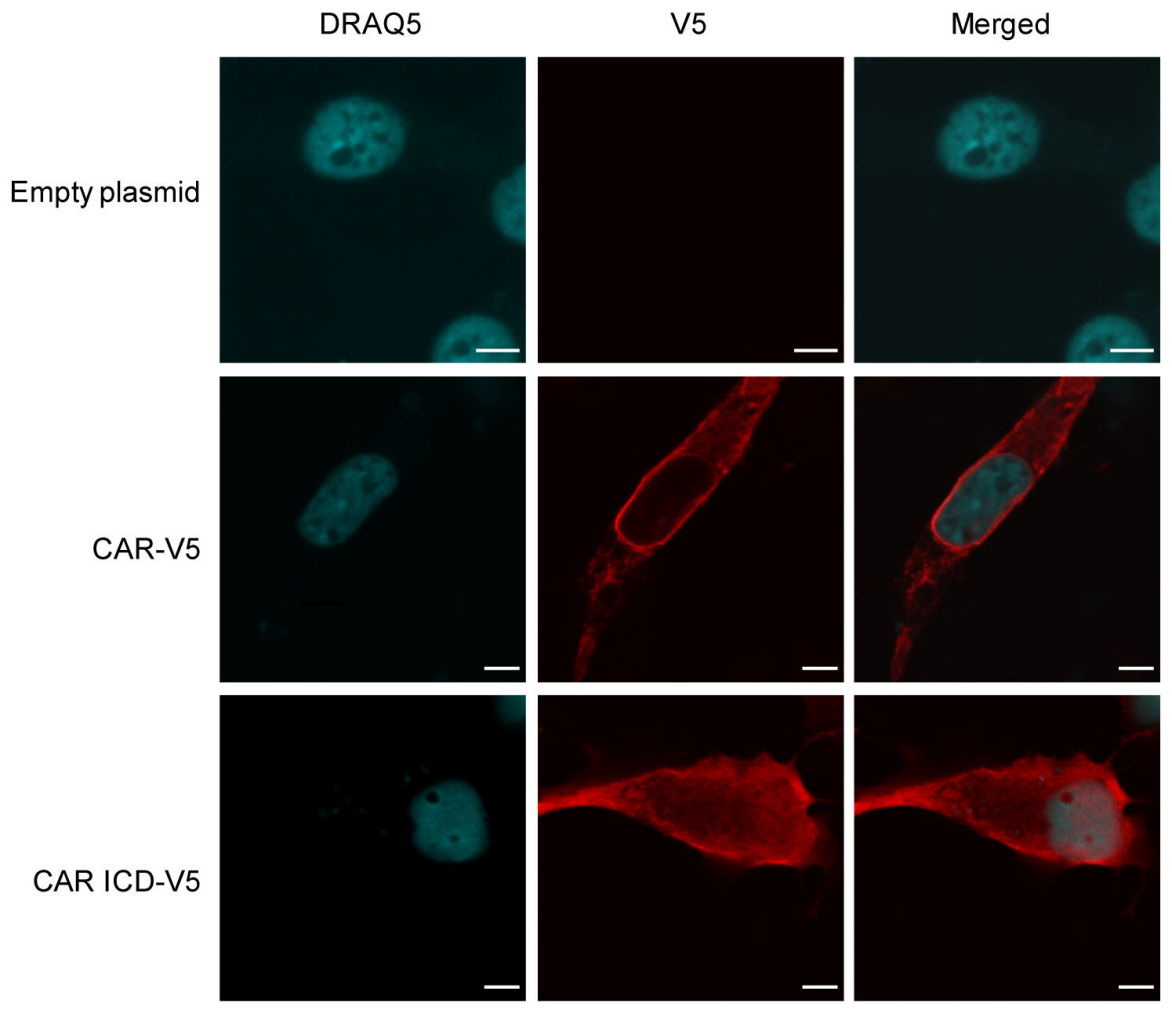
CAR’s intracellular domain (ICD) enters the nucleus. Immunofluorescence and confocal microscopy images showing the presence of CAR ICD in nuclei of U87-MG cells. U87-MG cells were transiently transfected with empty pcDNA3.1 V5/His plasmid, full-length CAR-V5 plasmid or with CAR ICD-V5 plasmid. Immunofluorescence staining was performed 24-48 hours post-transfection using anti-V5 tag antibody and Alexa Fluor 555 secondary antibody (red). Nuclei were stained with DRAQ5 (blue). Images were acquired with a confocal microscope (63x oil objective). Images are representative of at least 3 independent experiments. Scale bars: 5 µm.

Finally, as RIP of some cell surface proteins produces intracellular fragments that enter the nucleus, we investigated if that is the case for CAR. Nuclear immunoreactivity was observed with transient expression of full-length CAR-V5 in 293 cells (data not shown); however, the appearance of these speckles was rare. We generated a construct expressing the intracellular domain (amino acids 261-365 of murine CAR isoform 1) tagged at the C-terminus with V5, named CAR ICD-V5. U87-MG cells were transiently transfected with empty plasmid, full-length CAR-V5 or CAR ICD-V5, and immunofluorescence experiments were performed. Images were acquired using a confocal microscope. The ICD was readily detected in nuclei of U87 cells (Figure 10 and Figure S7). Similar results were obtained from experiments using 293A cells (data not shown). Thus, the intracellular domain of CAR is capable of nuclear entry.

We hypothesized that CAR CTF2 is not readily detectable in nuclei due to rapid proteasomal degradation of this fragment. Indeed, treatment of U87 CAR-V5 cells with the proteasome inhibitor epoxomicin increased levels of CTF2 and the ratio of CTF2 to CTF1 (Figure S8A and Figure S8B). Levels of free CAR ICD also accumulated in the presence of the proteasome inhibitor MG132 (Figure S8C). However, despite the improvement of CTF2 levels with epoxomicin, any nuclear CTF2 remained below detection level in epoxomicin-treated U87 CAR-V5 cells (data not shown).

## Discussion

A wide variety of cell surface proteins, including cell adhesion molecules, shed their ectodomains and also undergo RIP [25,29]. Here, we report that the cell adhesion molecule and virus receptor CAR is also subject to these processing events. CAR ECD shed from glioma cells ectopically expressing CAR as well as from developing neurons with endogenous CAR expression (Figure 1). In addition to this constitutive shedding, CAR ECD shed in a regulated fashion when cells were treated with the phorbol ester PMA (under chronic conditions) or with the calcium ionophore ionomycin (Figure 2), indicating an involvement of the PKC and calcium pathways, respectively, as has been demonstrated for a growing number of other cell surface proteins. The PKC and calcium signaling pathways are involved in cell migration, neuronal growth cone function and neurite outgrowth of developing neurons [46–49]. Regulation of CAR ECD shedding by these signaling pathways may thus have implications for CAR’s role as a cell adhesion molecule [6,8].

Using various approaches, we identified ADAM10 as the metalloprotease mediating constitutive and ionomycin-induced shedding of CAR, as well as shedding induced by high concentrations of PMA, from human glioma cells (Figure 3, Figure 4, Figure 5 and Figure 6). As ADAMs do not have consensus cleavage sites on substrates, we performed *in vitro* peptide digestion assays and mass spectrometry to determine the site of ADAM10 cleavage on CAR (Figure S4). The mutant MLRL AAAA stably expressed in HEK 293 cells did not shed its ECD into conditioned media (Figure 7B), indicating that amino acids 224-227 are important for CAR ECD shedding. Interestingly, deletion of a larger area consisting of amino acids 221-232 resulted in a mutant that readily shed its ECD (Figure 7B), perhaps due to a change in protein conformation that allowed the mutant receptor to be processed by ADAM10 or some other protease. While the mutants were expressed similarly to wild-type CAR at the cell surface in HEK 293 cells (Figure 7B), they were greatly diminished in expression at the surface of U251N cells (Figure S5D). The differences in cell surface expression levels of the mutants in HEK 293 and U251N cells may be due to differences in post-translational modification or protein folding.

Although ADAM10 is clearly a sheddase of CAR in our system, it is possible that other proteases, whether from the ADAM family or other metalloprotease families, are also capable of cleaving CAR’s ECD. For example, the cell adhesion molecule L1 is shed by both ADAM10 and ADAM17 although via different stimuli [26], and the hyaluronan receptor CD44 is shed by ADAM10, ADAM17 [50] and the matrix metalloproteinases MMP-9 [51] and MMP-14 [52]. Different proteases have been reported to mediate shedding of the same substrates in different cell types [53,54], suggesting redundancy in some cases.

CAR undergoes RIP via the γ-secretase complex (Figure 8) after ECD shedding (Figure 9 and Figure S6). Such a two-step process has been observed for other substrates of γ-secretase including L1 [26], protein-tyrosine kinase 7 (PTK7) [55] and the epidermal growth factor-like betacellulin precursor [56]. This sequential processing appears to be a general mechanism for γ-secretase substrates with permissive cytoplasmic and transmembrane domains [57], allowing for nicastrin, a member of the γ-secretase complex, to recognize RIP substrates [58].

As some intracellular domain products translocate to the nucleus following RIP [29], including Notch ICD [59] and even the ICD of ADAM10 [60], we investigated if that is the case for CAR. Indeed, transient expression of CAR ICD revealed that this portion of CAR enters the nucleus (Figure 10 and Figure S7). On the other hand, nuclear entry of CAR CTF2 generated via RIP was difficult to detect (data not shown), possibly due to its degradation by the proteasome (Figure S8). Degradation of RIP-generated intracellular domain fragments has been reported for other γ-secretase substrates such as Notch, syndecan-3, nectin-1α, p75, deleted in colorectal cancer (DCC) and members of the APP family [61–64]. However, RIP products, such as the Notch ICD, can nevertheless impart cellular effects at amounts below detection levels [65]. Another possible reason for the difficulty in detecting CAR CTF2 in the nucleus may be due to retention of the fragment at the plasma membrane via palmitoylation. Cysteine palmitoylation of CAR at amino acids 259 and 260 is required for proper cell surface targeting of the receptor [66]. These cysteines are the first two amino acids in CAR’s cytoplasmic domain. Since RIP of CAR likely occurs upstream of these residues within the transmembrane domain, perhaps the resulting CTF2 fragment remains tethered to the plasma membrane by palmitoylation. As palmitoylation is a dynamic and reversible modification [67,68], in this scenario a fraction of CTF2 fragments may be depalmitoylated and freed from the plasma membrane to enter the nucleus.

While ECD shedding and RIP may be mechanisms for CAR degradation and protein turnover, it is possible that these processing events can modulate physiological functions of CAR including its role as a cell adhesion molecule in the developing brain [6]. Shedding of the cell adhesion molecule neuroligin antagonizes spine formation of neuronal dendrites [69], while metalloprotease activity is required for neurite outgrowth mediated by L1 [26] and CHL1 [27]. Proteolysis of neural cell adhesion molecule (NCAM) in hippocampal neurons promotes their outgrowth [70,71], but NCAM proteolysis and addition of soluble NCAM ECD inhibits outgrowth of cortical neurons [72,73]. CAR mediates outgrowth of developing neurons [8], so its shedding may regulate this function as has been described for other neuronal cell adhesion molecules. It is also possible that CAR ICD, generated from RIP, may be involved in regulating neurite outgrowth. For example, RIP of the neurotrophin p75 receptor promotes neurite outgrowth, branching and number in dorsal root ganglion neurons, relieving inhibition by myelin-derived ligands [74], and promotes neurite outgrowth of tropomyosin receptor kinase family member A (TrkA)-expressing neurons in response to nerve growth factor (NGF) [75].

In polarized epithelial cells, CAR participates in the formation of tight junctions, where its expression is associated with lower cell permeability [9,76]. It also localizes to cell-cell contacts in non-polarized epithelial cells [9]. ADAM10 has been reported to localize mainly in adherens junctions, and to a limited extent in tight junctions, of polarized epithelial cells, and its proper targeting to adherens junctions promotes cell migration in a wound healing assay [77]. Perhaps ADAM10 or other metalloproteases can access CAR in tight junctions and shed its ECD, possibly downregulating CAR-mediated tight junction integrity.

Finally, proteolysis may play a role in regulation of CAR’s function as a virus receptor. One can imagine that in a situation where proteolysis of CAR at the cell surface is upregulated, for example, via the PKC or calcium signaling pathways or by increased activity of ADAM10, host cells would be rendered less susceptible to infection by Coxsackievirus and adenovirus serotypes that require binding to CAR’s extracellular domain.

In conclusion, the characterization of shedding and RIP of CAR presented in this work promote our understanding of the cell adhesion molecule and virus receptor CAR. Future work will reveal what roles, if any, are played by these proteolytic cleavages in the function of CAR.

## Supporting Information

Figure S1 **A fragment of CAR consisting of part of its extracellular domain is shed into media of U87 cells and cannot be detected with an anti-C terminus antibody.** A 32 kDa fragment of CAR was released into conditioned media of U87 CAR cells upon 4 hours of treatment with 1 µM PMA. This fragment was recognized by anti-CAR extracellular domain antibody 2240 (A), but not by antibody RP291 raised against CAR intracellular domain (B).
(TIF)

Figure S2 **PMA treatment leads to CAR phosphorylation.** U87 CAR cells were treated for 4 hours with 1 µM PMA vs. DMSO vehicle, and cell lysates were collected. Calf intestinal phosphatase (CIP) treatment of lysates abolished the appearance of the higher molecular weight species of full-length CAR obtained with PMA treatment, indicating that PMA causes a post-translational modification of full-length CAR in the form of phosphorylation. Western blotting was performed with the anti-CAR N-terminus antibody 2240.
(TIF)

Figure S3 **Real-time quantitative PCR for verification of knockdown of ADAM10 mRNA levels.** U87 CAR stable cell lines infected with lentivirus containing control (anti-eGFP) shRNA or anti-ADAM10 (#6675 or #6676) shRNA were generated. RNA was isolated from these cells, followed by reverse transcription to cDNA and real-time PCR in triplicates to quantify ADAM10, ADAM17 and GAPDH expression levels. The two anti-ADAM10 shRNA sequences #6675 and #6676 successfully knocked down mRNA levels of ADAM10 compared to control shRNA without affecting expression levels of the related family member ADAM17.
(TIF)

Figure S4 **Mapping the sites of ECD cleavage on CAR.** A 20-amino acid peptide (VGSDQCMLRLDVVPPSNRAG) representing the juxtamembrane region in CAR ECD was digested with recombinant human ADAM10 at 37°C for 4 or 16 hours, along with 3 controls (recombinant ADAM10 only, 16 hours; peptide only, 16 hours; peptide and recombinant ADAM10; 0 hours). Samples were analyzed by MALDI-MS. Two unique peaks (shaded grey) at (A) 1008 m/z and (B) 1393 m/z were found that were not present in the 3 controls. Further analysis was done with MS/MS in order to deduce the identities of the amino acids in each peptide fragment. These results represent 2 independent experiments.
(TIF)

Figure S5 **Characterization of CAR ECD mutants in human glioma U251N cells.** (A) Stable U251N cell lines of mock (empty vector), wild-type CAR, and 3 mutants (MLAA, RL AA and Δ221-232) were generated. Constitutive shedding of CAR and the mutants was assayed. Mutating pairs of amino acids to alanine (MLAA and RLAA) led to a decrease in CAR ECD shedding. However, this inhibition was reversed in subsequent cell passages. Deletion of 12 amino acids (Δ221-232) containing the potential area of ECD cleavage resulted in a mutant that still shed. (B) A mutant CAR was generated in which amino acids 224-227 were changed to alanine residues (MLRL AAAA), and was stably expressed in U251N cells. Shedding of this mutant was completely abrogated. Cell surface biotinylation experiments (panels C and D) revealed that all the mutants were expressed at much lower levels at the surface of U251N cells compared to wild-type CAR.
(TIF)

Figure S6 **GM6001 treatment results in a decrease in CAR CTF1 and CTF2 levels.** U87 cells stably expressing CAR with a C-terminal V5 tag (CAR-V5) were treated with 25 µM of the metalloprotease inhibitor GM6001 or its negative control for 4 hours. Conditioned media and lysates were collected as described, and Western blotting was performed with the anti-CAR N-terminus antibody 2240 (for conditioned media) and anti-V5 tag antibody (for lysates). GM6001 treatment abrogated CAR ECD shedding as expected. There was a small decrease in levels of both CAR CTF1 and CTF2 with GM6001 treatment.
(TIF)

Figure S7 **Z stack images of a U87 cell transiently expressing CAR ICD.** Confocal microscopy Z stack images were acquired of a U87 cell transiently expressing V5-tagged CAR ICD (red = anti-V5). Shown are 20 slices representing a total thickness of 6.59 µm. Scale bar: 5 µm.
(TIF)

Figure S8 **CAR ICD is subject to proteasomal degradation.** (A) U87 CAR-V5 cells were treated for 16 hours with the proteasome inhibitor epoxomicin (1 µM or 5 µM) vs. DMSO vehicle. Shown is a representative Western blot performed using antibody raised against the V5 tag. (B) CTF1 and CTF2 band intensities were quantified from Western blots, and ratios of CTF2/CTF1 were calculated. The graph represents mean CTF2/CTF1 ratios obtained from 3 independent experiments (n=3 per group). One-way ANOVA with Bonferroni post-test, * = p < 0.05. (C) U87 cells transiently expressing V5-tagged CAR ICD were treated overnight with the proteasome inhibitor MG132 (25 µM) or DMSO vehicle control. Samples were analyzed by Western blotting for GAPDH and the V5 tag. Treatment with MG132 led to an accumulation of CAR ICD levels.
(TIF)
