# Supplementary figures and images for: The Coxsackievirus and Adenovirus Receptor (CAR) Undergoes Ectodomain Shedding and Regulated Intramembrane Proteolysis (RIP)
Source: PLoS One. 2013 Aug 28;8(8):e73296. doi: 10.1371/journal.pone.0073296 (PMC3756012; doi:10.1371/journal.pone.0073296)

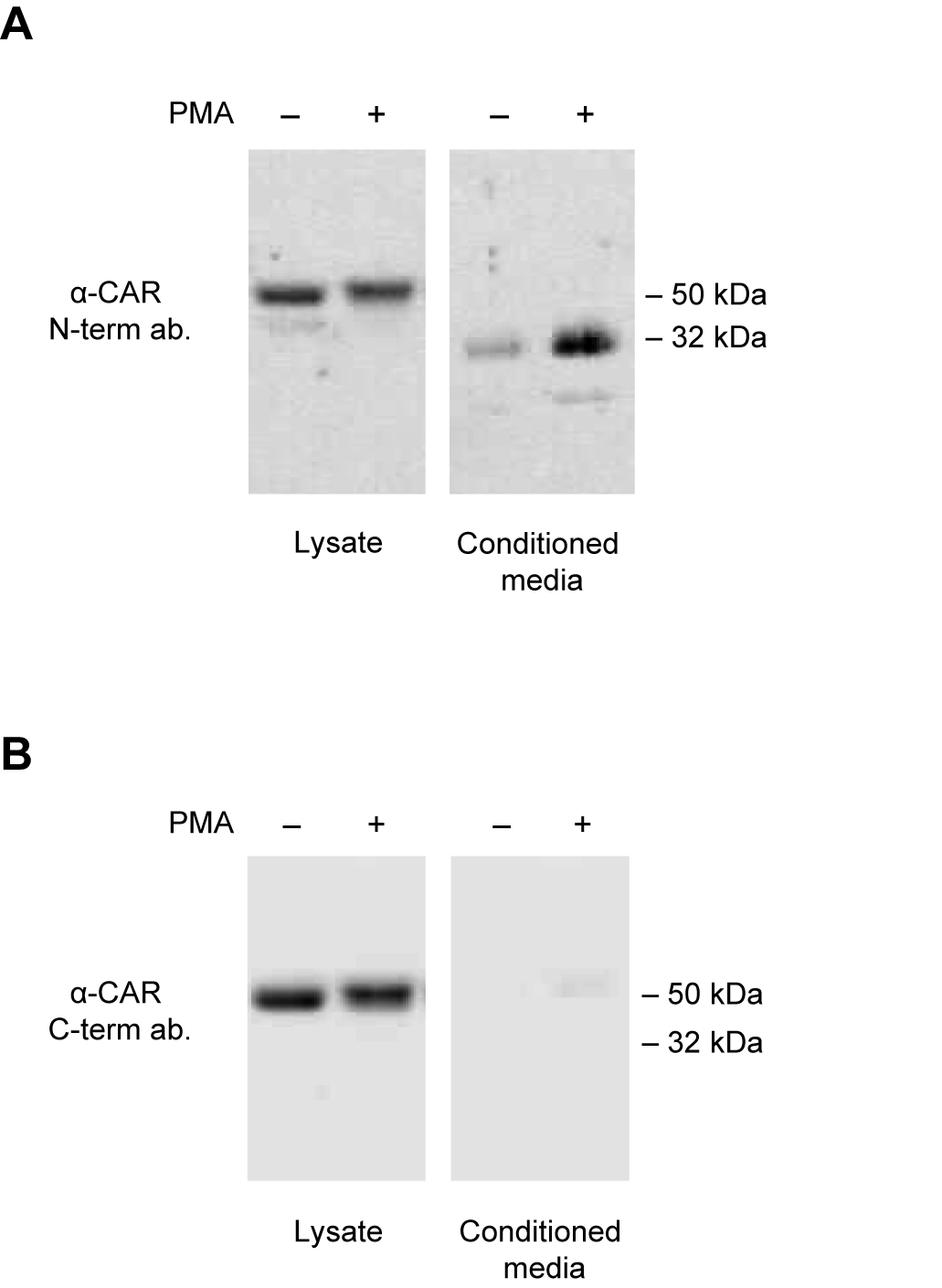

Supplement: Figure S1 — A fragment of CAR consisting of part of its extracellular domain is shed into media of U87 cells and cannot be detected with an anti-C terminus antibody. A 32 kDa fragment of CAR was released into conditioned media of U87 CAR cells upon 4 hours of treatment with 1 µM PMA. This fragment was recognized by anti-CAR extracellular domain antibody 2240 (A), but not by antibody RP291 raised against CAR intracellular domain (B). (TIF) [file pone.0073296.s001.tif]

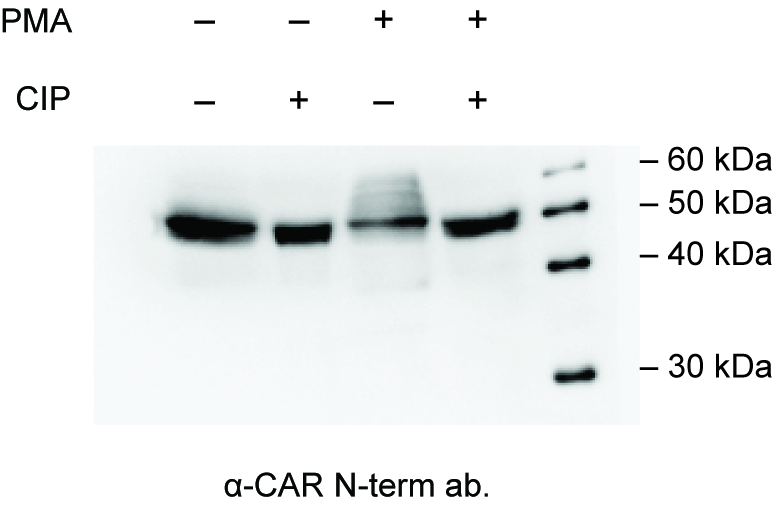

Supplement: Figure S2 — PMA treatment leads to CAR phosphorylation. U87 CAR cells were treated for 4 hours with 1 µM PMA vs. DMSO vehicle, and cell lysates were collected. Calf intestinal phosphatase (CIP) treatment of lysates abolished the appearance of the higher molecular weight species of full-length CAR obtained with PMA treatment, indicating that PMA causes a post-translational modification of full-length CAR in the form of phosphorylation. Western blotting was performed with the anti-CAR N-terminus antibody 2240. (TIF) [file pone.0073296.s002.tif]

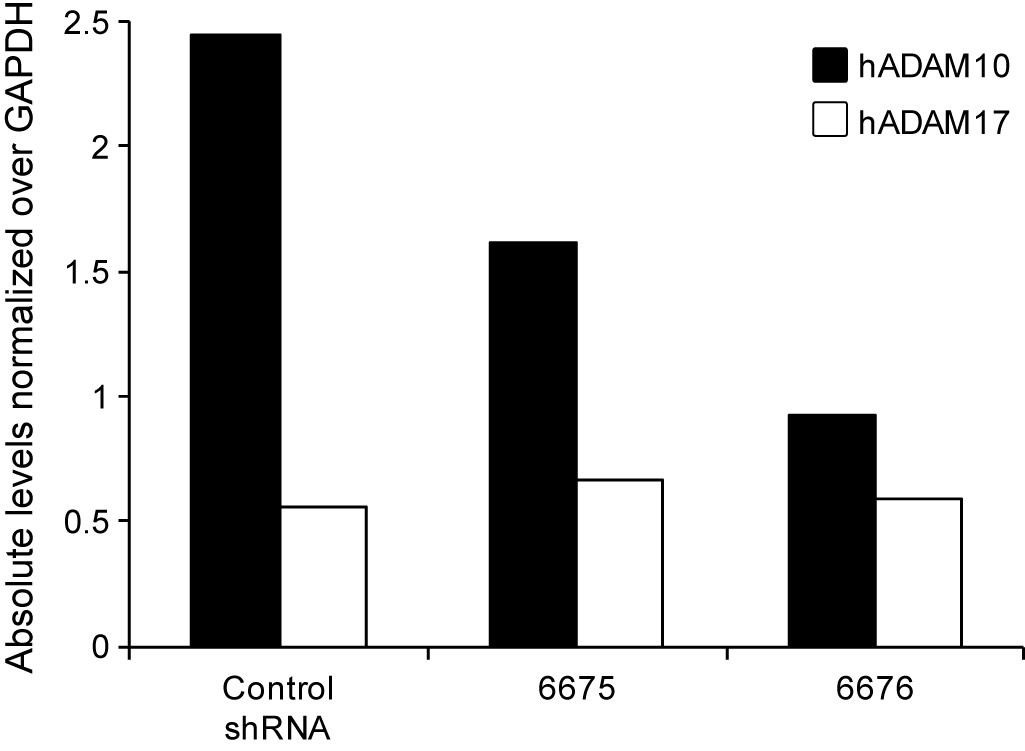

Supplement: Figure S3 — Real-time quantitative PCR for verification of knockdown of ADAM10 mRNA levels. U87 CAR stable cell lines infected with lentivirus containing control (anti-eGFP) shRNA or anti-ADAM10 (#6675 or #6676) shRNA were generated. RNA was isolated from these cells, followed by reverse transcription to cDNA and real-time PCR in triplicates to quantify ADAM10, ADAM17 and GAPDH expression levels. The two anti-ADAM10 shRNA sequences #6675 and #6676 successfully knocked down mRNA levels of ADAM10 compared to control shRNA without affecting expression levels of the related family member ADAM17. (TIF) [file pone.0073296.s003.tif]

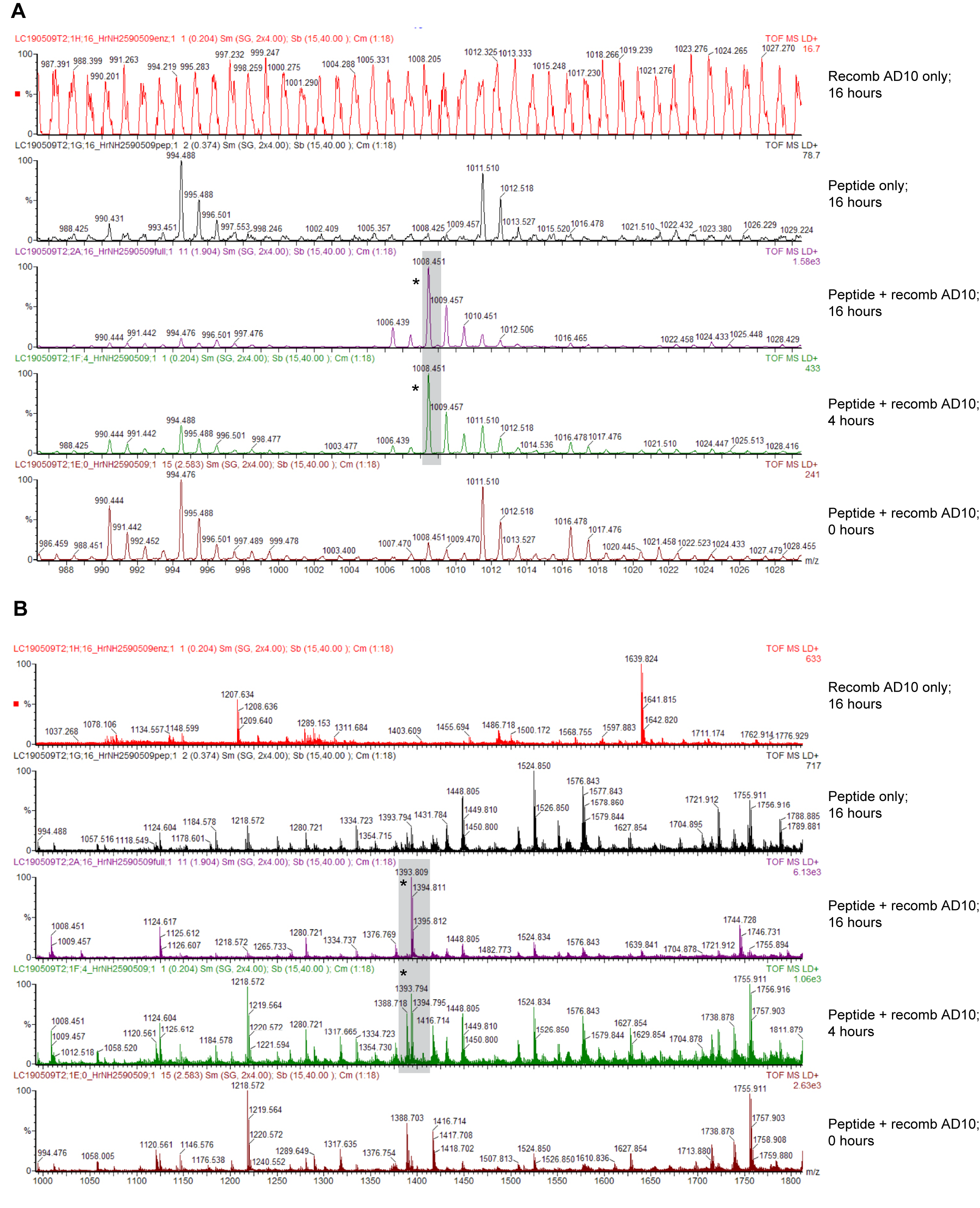

Supplement: Figure S4 — Mapping the sites of ECD cleavage on CAR. A 20-amino acid peptide (VGSDQCMLRLDVVPPSNRAG) representing the juxtamembrane region in CAR ECD was digested with recombinant human ADAM10 at 37°C for 4 or 16 hours, along with 3 controls (recombinant ADAM10 only, 16 hours; peptide only, 16 hours; peptide and recombinant ADAM10; 0 hours). Samples were analyzed by MALDI-MS. Two unique peaks (shaded grey) at (A) 1008 m/z and (B) 1393 m/z were found that were not present in the 3 controls. Further analysis was done with MS/MS in order to deduce the identities of the amino acids in each peptide fragment. These results represent 2 independent experiments. (TIF) [file pone.0073296.s004.tif]

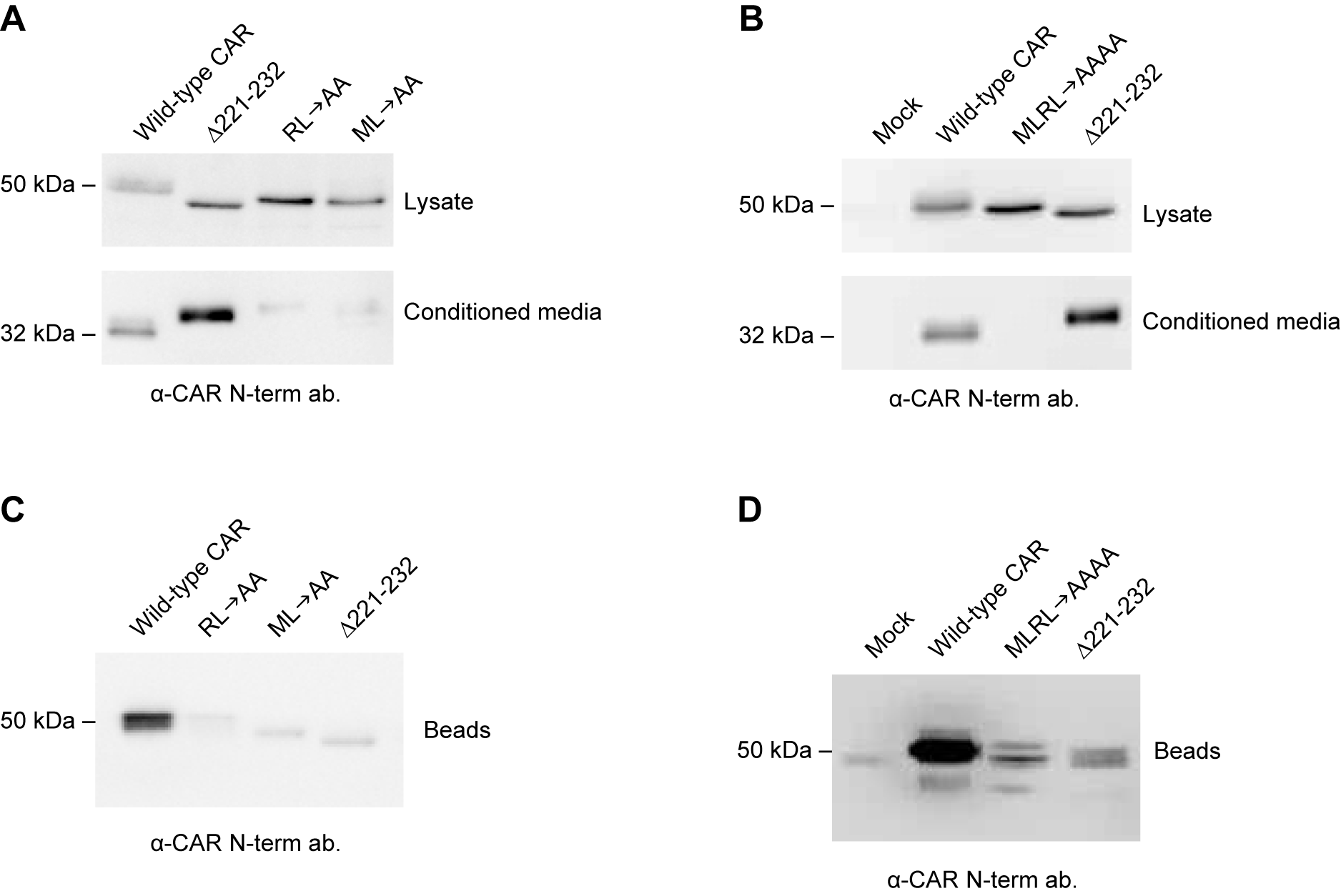

Supplement: Figure S5 — Characterization of CAR ECD mutants in human glioma U251N cells. (A) Stable U251N cell lines of mock (empty vector), wild-type CAR, and 3 mutants (MLAA, RL AA and Δ221-232) were generated. Constitutive shedding of CAR and the mutants was assayed. Mutating pairs of amino acids to alanine (MLAA and RLAA) led to a decrease in CAR ECD shedding. However, this inhibition was reversed in subsequent cell passages. Deletion of 12 amino acids (Δ221-232) containing the potential area of ECD cleavage resulted in a mutant that still shed. (B) A mutant CAR was generated in which amino acids 224-227 were changed to alanine residues (MLRL AAAA), and was stably expressed in U251N cells. Shedding of this mutant was completely abrogated. Cell surface biotinylation experiments (panels C and D) revealed that all the mutants were expressed at much lower levels at the surface of U251N cells compared to wild-type CAR. (TIF) [file pone.0073296.s005.tif]

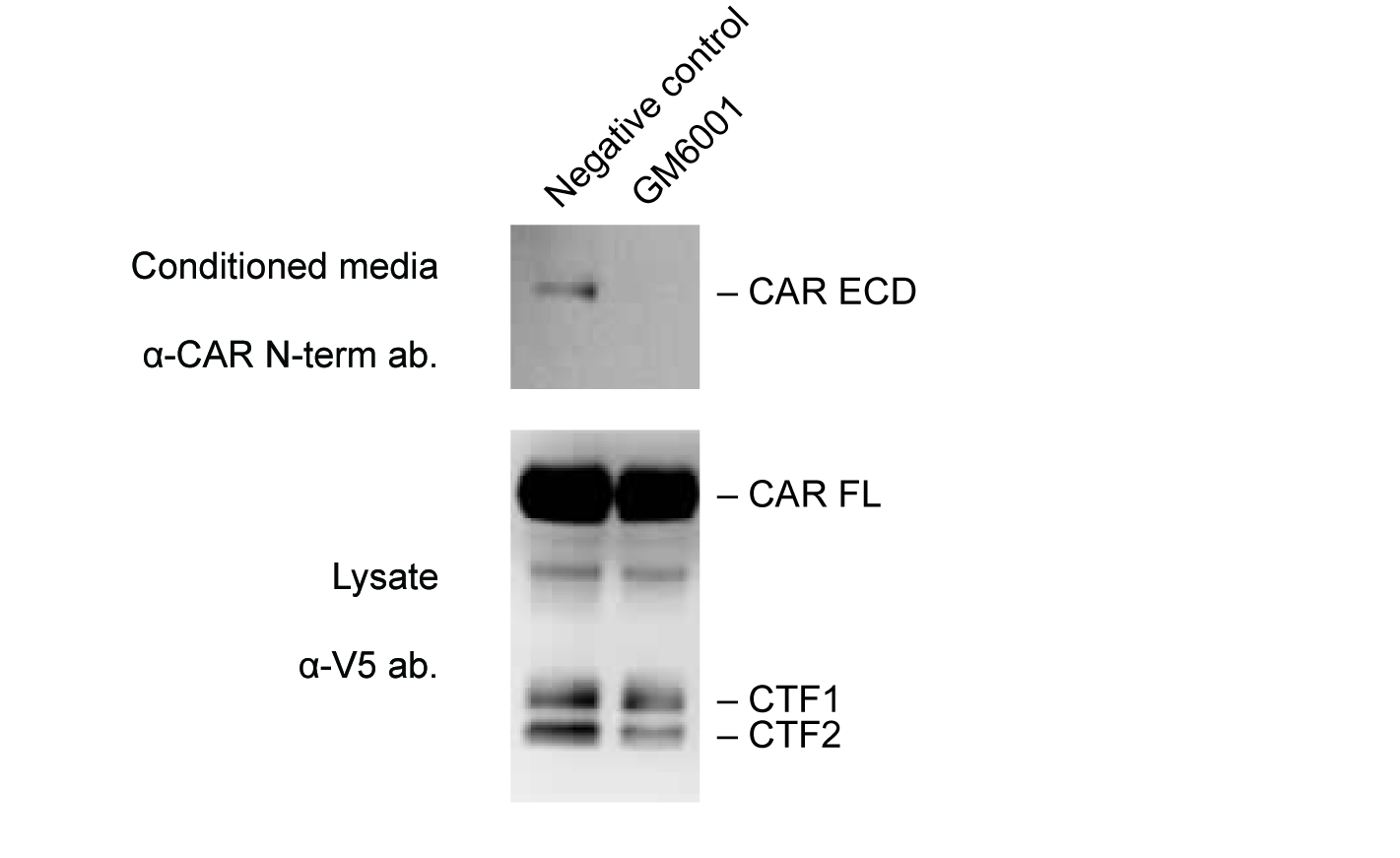

Supplement: Figure S6 — GM6001 treatment results in a decrease in CAR CTF1 and CTF2 levels. U87 cells stably expressing CAR with a C-terminal V5 tag (CAR-V5) were treated with 25 µM of the metalloprotease inhibitor GM6001 or its negative control for 4 hours. Conditioned media and lysates were collected as described, and Western blotting was performed with the anti-CAR N-terminus antibody 2240 (for conditioned media) and anti-V5 tag antibody (for lysates). GM6001 treatment abrogated CAR ECD shedding as expected. There was a small decrease in levels of both CAR CTF1 and CTF2 with GM6001 treatment. (TIF) [file pone.0073296.s006.tif]

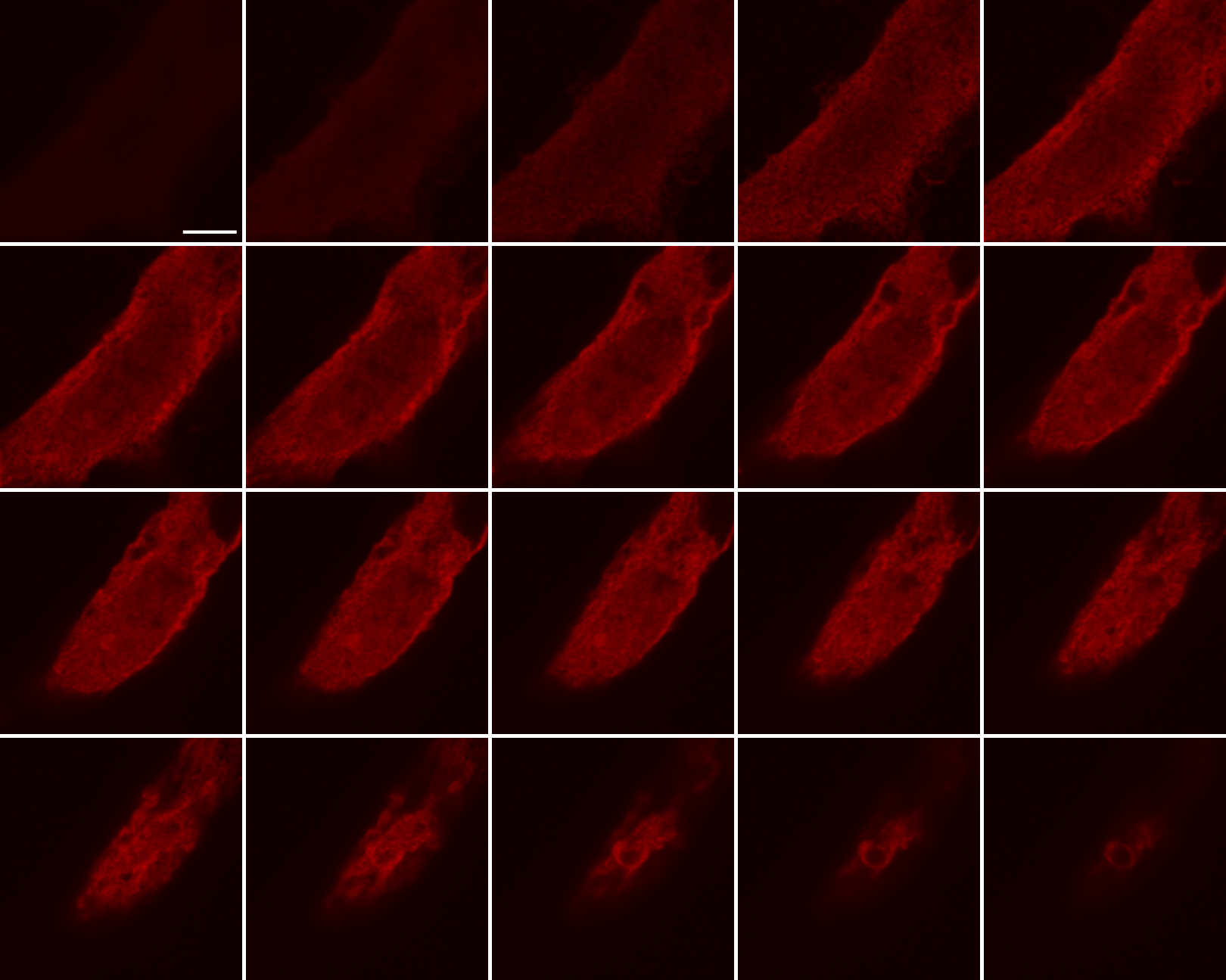

Supplement: Figure S7 — Z stack images of a U87 cell transiently expressing CAR ICD. Confocal microscopy Z stack images were acquired of a U87 cell transiently expressing V5-tagged CAR ICD (red = anti-V5). Shown are 20 slices representing a total thickness of 6.59 µm. Scale bar: 5 µm. (TIF) [file pone.0073296.s007.tif]

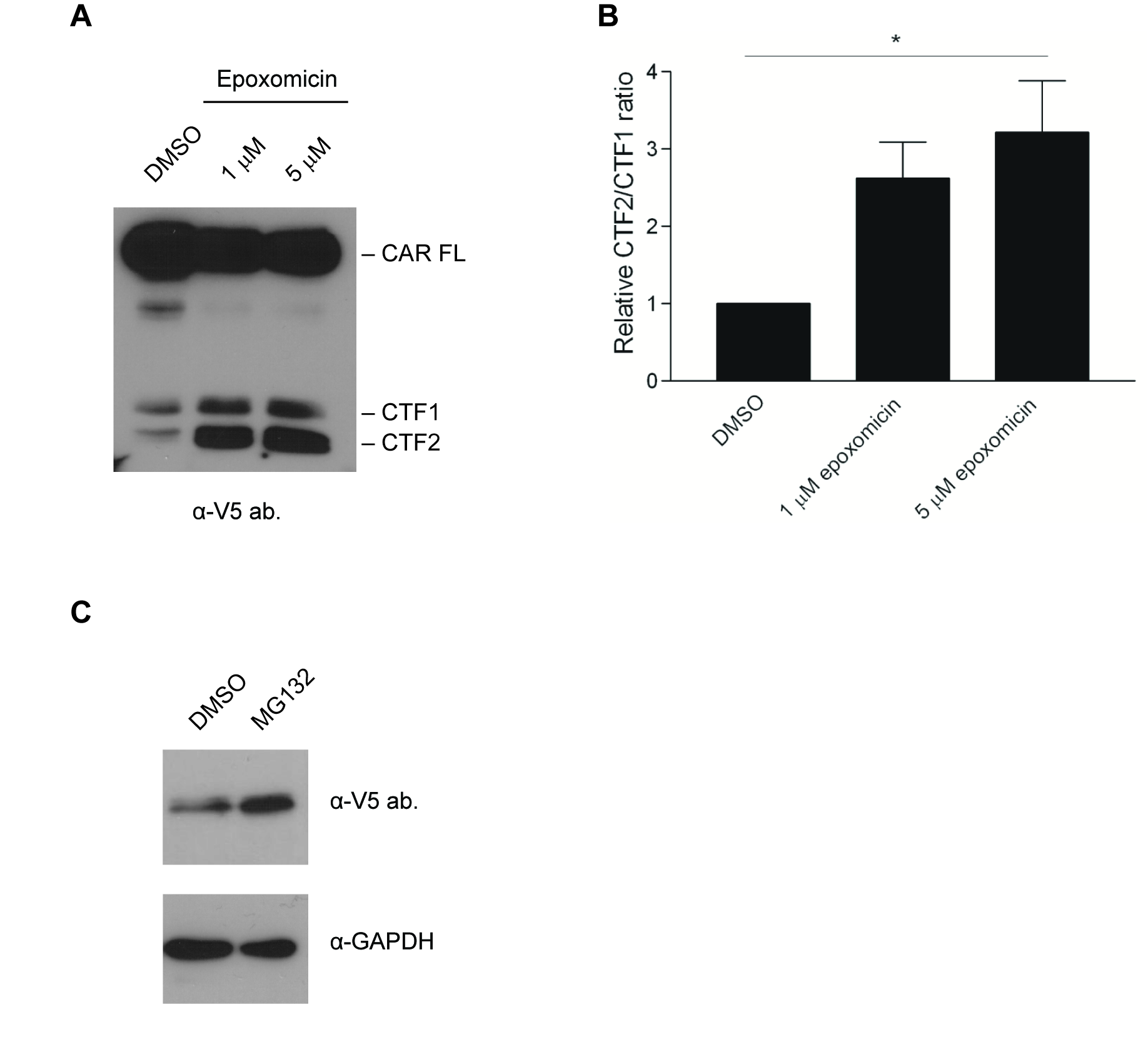

Supplement: Figure S8 — CAR ICD is subject to proteasomal degradation. (A) U87 CAR-V5 cells were treated for 16 hours with the proteasome inhibitor epoxomicin (1 µM or 5 µM) vs. DMSO vehicle. Shown is a representative Western blot performed using antibody raised against the V5 tag. (B) CTF1 and CTF2 band intensities were quantified from Western blots, and ratios of CTF2/CTF1 were calculated. The graph represents mean CTF2/CTF1 ratios obtained from 3 independent experiments (n=3 per group). One-way ANOVA with Bonferroni post-test, * = p < 0.05. (C) U87 cells transiently expressing V5-tagged CAR ICD were treated overnight with the proteasome inhibitor MG132 (25 µM) or DMSO vehicle control. Samples were analyzed by Western blotting for GAPDH and the V5 tag. Treatment with MG132 led to an accumulation of CAR ICD levels. (TIF) [file pone.0073296.s008.tif]
